# Characterization of soil bacterial, archaeal and fungal communities inhabiting archaeological human-impacted layers at Monte Iato settlement (Sicily, Italy)

**DOI:** 10.1038/s41598-018-20347-8

**Published:** 2018-01-30

**Authors:** José A. Siles, Birgit Öhlinger, Tomas Cajthaml, Erich Kistler, Rosa Margesin

**Affiliations:** 1 Institute of Microbiology, University of Innsbruck, Technikerstrasse 25, A-6020 Innsbruck, Austria; 2 Institute of Archaeologies, University of Innsbruck, Langer Weg 11, A-6020 Innsbruck, Austria; 3 Institute of Microbiology, Academy of Sciences of the Czech Republic, v.v.i., Vídeňská 1083, CZ-142 20 Prague 4, Czech Republic; 4 Institute for Environmental Studies, Faculty of Science, Charles University in Prague, Benatska 2, CZ-128 01 Prague 2, Czech Republic

## Abstract

Microbial communities in human-impacted soils of ancient settlements have been proposed to be used as ecofacts (bioindicators) of different ancient anthropogenic activities. In this study, bacterial, archaeal and fungal communities inhabiting soil of three archaic layers, excavated at the archaeological site on Monte Iato (Sicily, Italy) and believed to have been created in a chronological order in archaic times in the context of periodic cultic feasts, were investigated in terms of (i) abundance (phospholipid fatty acid (PLFA) analysis and quantitative PCR)), (ii) carbon(C)-source consumption patterns (Biolog-Ecoplates) and (iii) diversity and community composition (Illumina amplicon sequencing). PLFA analyses demonstrated the existence of living bacteria and fungi in the soil samples of all three layers. The upper layer showed increased levels of organic C, which were not concomitant with an increment in the microbial abundance. In taxonomic terms, the results indicated that bacterial, archaeal and fungal communities were highly diverse, although differences in richness or diversity among the three layers were not detected for any of the communities. However, significantly different microbial C-source utilization patterns and structures of bacterial, archaeal and fungal communities in the three layers confirmed that changing features of soil microbial communities reflect different past human activities.

## Introduction

The coming together of archaeology and microbiology promises to revolutionize the analysis and interpretation of archaeological deposits^1^. In this way, the few existing works studying the microbial communities associated with archaeological samples such as dental calculi^2^, bones^3^, different kinds of organic remains^4^, anthropogenically-impacted soils^5–8^ and mud^6,9^ have confirmed the efficacy of this new direction of analysis in archaeology that may impact on the formulation of theories and our understanding of past events^1^.

In the context of the archaeological study of soils at ancient settlements, microbial communities can be used as precise indicators of ancient anthropogenic activities during soil formation in the distant past^10^. It has been suggested that changing features of microbial communities associated with archaeological human-impacted soil layers can be considered as a kind of archive of both environmental and anthropogenic conditions during the period of their formation, adding precise information about the nature of specific activities in the past. This is based, firstly, on the idea that the anthropogenic factor is most likely the crucial one in soil formation within a settlement, and secondly, on the fact that microorganisms are an integrated soil component, highly sensitive to environmental changes and able to effectively respond to changing environmental conditions^11^. Likewise, soil microbial communities are able to survive long periods of time under adverse environmental conditions through retreating into a dormant state^12^. Therefore, since microorganisms can specifically react to the input of nutrients in the form of organic substances, human activities changing the intensity and mode of those inputs may be reflected in soil microbial communities in quantitative and qualitative terms^11^. Former studies have already used microbial communities as bioindicators, termed as ecofacts by Margesin, *et al*.^5^, of human activities in the past. Soil fungal communities inhabiting anthropogenically transformed layers of medieval Russian settlements differed from those associated with non-impacted surrounding soils^13^. Studies at another medieval Russian settlement showed how spatial variations in the presence of keratin-decomposing fungi in sediments of streets, living-floors in and outside houses and from wall-filling revealed differences in the activities and the intensity of anthropogenic impact^8^. More recently, Peters, *et al*.^11^, in a case study from a Late Bronze Age settlement in the North Caucasus (Russia), confirmed that both activities and abundances of soil microbial communities change among the different layers analyzed depending on the intensity and the character of past human activities. Notwithstanding all these works, there is still a lack of surveys considering overall microbial (bacterial, archaeal and fungal) communities in terms activity, abundance, diversity and composition at archaeological sites, in general, and at anthropogenically transformed sites as a tool to differentiate layers that cannot be perceived by macroscopic analysis, in particular.

The archaeological settlement on Monte Iato is located in the inland of Sicily (Italy), about 30 km south-west of Palermo (37°58′01.9″N, 13°11′44.0″E), and was scattered over the slightly sloping 40 hectare wide plateau of the hill. Its strategic location for defence purposes is typical for Iron Age settlements of the Sicilian hinterland^14–16^. Details regarding the history of this settlement have previously been reported^5^. The position and the structure of the settlements followed the common pattern of so-called archaeological compounds (hamlet-like structures), which were loosely spread over this settled area. These compounds consisted of several rectangular, round and oval-shaped buildings that were mostly grouped around an open space. Each compound was occupied by an extended family and had its special family-internal cult area, normally comprising of a round building, where sacrificial meals took place within the family or clan. Parts of a protohistoric dwelling of central ritual importance have been excavated in the last years in one of these compounds^17–19^. The cultic character is attested by two ritual deposits outside and within the building which have been created in the course of the 6th century BC^19,20^.

22 m to the east of this structure, three clearly separated layers of stone packings, laying on top of each other and composed of stones and soil, were discovered in September 2015. Between the stones of the individual packings, numerous incised sherds and fragments of matt-painted pottery were found. Additionally, noteworthy amounts of bone fragments with hack and slaughter marks came to light^21^. Especially the latter are indicative of the formation of these layers of stone packings in the context of sacrificial feasts. A rim fragment found underneath, directly reminiscent of amphora-like pithoi from the Necropolis of Himera, shows that the three layers cannot be dated before 575–50 BC^22^. The layers of stone packings were clearly separated by thin layers of alluvial material. These alluvial separation layers within the otherwise homogenous material demonstrate that the different layers of stone packings were lying open for some time and therefore proof their formation in the context of periodically celebrated sacrificial feasts, which has not yet been documented before; maybe the packings were meant to compress the clayey ground, so that feast participants from afar would be able to erect mobile dwellings during the second and third quarters of the 6th century BC^21^.

The objectives of this study were: (i) to characterize soil microbial (bacterial, archaeal and fungal) communities in the three layers of stone packings in terms of abundance, physiological profiles, diversity and taxonomic composition, and (ii) to evaluate whether characteristics of soil microbial communities in the three layers, formed chronologically on top of each other, are significantly different. We hypothesize (i) that bacterial, archaeal and fungal communities present in the studied occupation layers are characterized by a wide diversity and (ii) that the structure of the microbial communities differs among the layers as a consequence of different cultic events. Thus, changing microbial features can serve as ecofacts in these human-impacted layers created in the context of cultic events on Archaic Monte Iato and give evidence of ancient human behavior and ritual praxis. This work complements our previous study on the characterization of soil bacterial and fungal communities at another location of the same archaeological settlement differently impacted by ancient human activities^5^.

## Results

### Soil physicochemical properties

The studied soil samples were characterized by close to neutral pH values and low levels of nutrients (Table 1). Soil samples of the upper layer A14 contained significantly higher contents of SOM (soil organic matter) and TOC (total organic carbon) and higher TOC/N ratios than soil samples of the lower layers A16 and A18 (Table 1).

**Table 1:** Physicochemical properties, microbial abundances and potential bacterial respiration determined in soil samples of the studied layers A14 (upper), A16 (intermediate) and A18 (lower). For each variable, mean values followed by different letters are significantly different (p ≤ 0.05).

	**A14**	**A16**	**A18**
**Soil physicochemical properties**			
pH	6.5–7.0a	6.5–7.0a	6.5–7.0a
SOM (%)^1^	3.03b	2.19a	2.43ab
TOC (%)^2^	1.76b	1.27a	1.41ab
N (%)	0.07a	0.08a	0.09a
TOC/N	24.04b	15.41a	15.12a
**PLFA-based microbial abundance**			
Total abundance (μg PLFA g^−1^ dry soil)	515.72a	387.60a	411.25a
Bacteria (B) (μg PLFA g^−1^ dry soil)	289.22a	180.24a	130.95a
Actinobacteria (μg PLFA g^−1^ dry soil)	59.09a	36.49a	20.59a
Fungi (F) (μg PLFA g^−1^ dry soil)	18.93a	26.09a	26.75a
F/B	0.07a	0.15ab	0.20b
**qPCR-based microbial abundance**			
Bacteria (B) (16S rRNA gene copy number g^−1^ dry soil)	1.45 × 10^7^a	1.52 × 10^7^a	1.62 × 10^7^a
Archaea (A) (16S rRNA gene copy number g^−1^ dry soil)	8.70 × 10^5^a	8.85 × 10^5^a	1.32 × 10^6^b
Fungi (F) (18S rRNA gene copy number g^−1^ dry soil)	2.25 × 10^4^a	3.57 × 10^4^a	8.25 × 10^4^a
A/B	0.83a	0.83a	0.85a
F/B	0.59a	0.63a	0.62a
**Potential bacterial respiration**			
*gtlA* (gene copy number g^−1^ dry soil)	7.73 × 10^3^b	4.57 × 10^3^a	3.76 × 10^3^a

^1^SOM, soil organic matter.

^2^TOC, total organic carbon.

### Microbial abundance

PLFA analyses demonstrated the existence of living microbial biomass in all soil samples; however, significant differences in terms of total, bacterial, actinobacterial or fungal biomass among the layers were not detected (Table 1). Based on qPCR-based microbial abundance (living and non-living microorganisms), soil samples of the lower layer A18 contained significantly higher archaeal 16S rRNA gene copy numbers respect to soil samples of the intermediate and upper layer. Neither copy numbers for bacterial and fungal communities nor the ratios A/B and F/B differed significantly among the soil samples of all three layers. The gene *gtlA*, as a measurement of potential aerobic bacterial respiration, was detected in all three layers and its abundance was significantly highest in the upper layer A14 (Table 1).

### Community level physiological profiles

**Figure 1:**
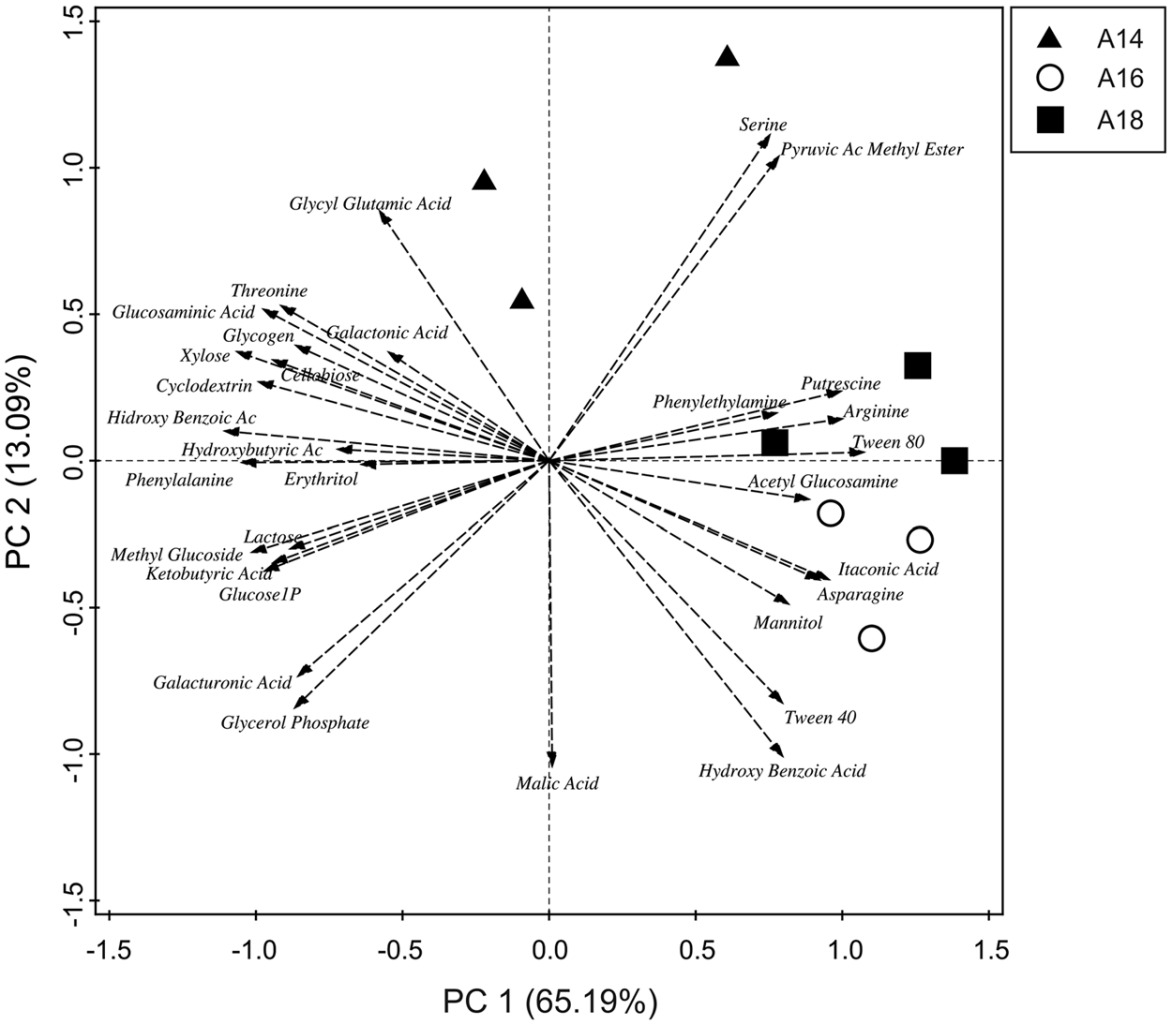
Principal component analysis (PCA) biplot of community level physiological profiles (CLPPs) found in soil samples of the studied layers A14 (upper), A16 (intermediate) and A18 (lower). Percent variability explained by each principal component is shown in parentheses after each axis legend.

The number of substrates oxidized (richness) did not significantly differ among the soil samples in the three layers, although Shannon index and evenness were higher in the intermediate layer A16 respect to the upper layer A14 (Table S1). Global and pairwise PERMANOVA (permutational multivariate analysis of variance) and ANOSIM (analysis of similarity) showed that C-source oxidation patterns significantly differed among the three layers (Table 2). SIMPER (similarity percentage) analysis evidenced that these differences were weighted heavily towards a few C sources, with the top 10 comprising 62.2% of the dissimilarity among layers (Table S2). The main C sources responsible for the differences among layers were: L-serine, pyruvic acid methyl ester and 4-hydroxy benzoic acid (oxidized to a higher extent in the upper layer A14); Tween 40, L-asparagine, D-mannitol and itaconic acid (preferably consumed in the intermediate layer A16); as well as Tween 80, L-arginine and putrescine (highest oxidation rates in the lower layer A18) (Fig. 1, Table S2). SIMPER pairwise comparisons demonstrated that dissimilarity between soil samples of the intermediate and lower layers (37.5%) was much lower than that between soil samples of the upper and the intermediate (74.1%) or lower layer (75.7%). This was evidenced in PCA (principal component analysis) since soil samples of the intermediate layer A16 and the lower layer A18 clustered together over PC 1 (Fig. 1).

**Table 2:** Results of global (considering the three layers) and pairwise (considering each pair of layers) PERMANOVA (permutational analysis of variance) and ANOSIM (analysis of similarities) of community level physiological profiles (CLPPs) and OTU-based bacterial, archaeal and fungal community structures found in the studied layers A14 (upper), A16 (intermediate) and A18 (lower). F and R values in bold denote statistical significance (p ≤ 0.05).

	**PERMANOVA**		**ANOSIM**	
	F	*p-value*	R	*p-value*
**Community level physiological profiles**				
A14 *vs*. A16 *vs*. A18	**5.213**	0.0001	**0.324**	0.0001
A14 *vs*. A16	**7.151**	0.0001	**0.398**	0.0001
A14 *vs*. A18	**4.468**	0.0002	**0.371**	0.0004
A16 *vs*. A18	**2.779**	0.0007	**0.238**	0.0007
**Bacterial community structure**				
A14 *vs*. A16 *vs*. A18	**5.384**	0.0002	**0.653**	0.0001
A14 *vs*. A16	**4.667**	0.0180	**0.511**	0.0059
A14 *vs*. A18	**5.787**	0.0008	**0.506**	0.0063
A16 *vs*. A18	**6.186**	0.0022	**0.991**	0.0003
**Archaeal community structure**				
A14 *vs*. A16 *vs*. A18	**6.212**	0.0094	**0.434**	0.0001
A14 *vs*. A16	4.797	0.0628	**0.298**	0.0038
A14 *vs*. A18	**7.673**	0.0196	**0.469**	0.0029
A16 *vs*. A18	**6.193**	0.0021	**0.732**	0.0023
**Fungal community structure**				
A14 *vs*. A16 *vs*. A18	**2.781**	0.0001	**0.283**	0.0003
A14 *vs*. A16	**3.471**	0.0024	**0.306**	0.0079
A14 *vs*. A18	**1.959**	0.0074	**0.196**	0.0416
A16 *vs*. A18	**3.167**	0.0058	**0.370**	0.0050

### Taxonomic composition of bacterial, archaeal and fungal communities

**Figure 2:**
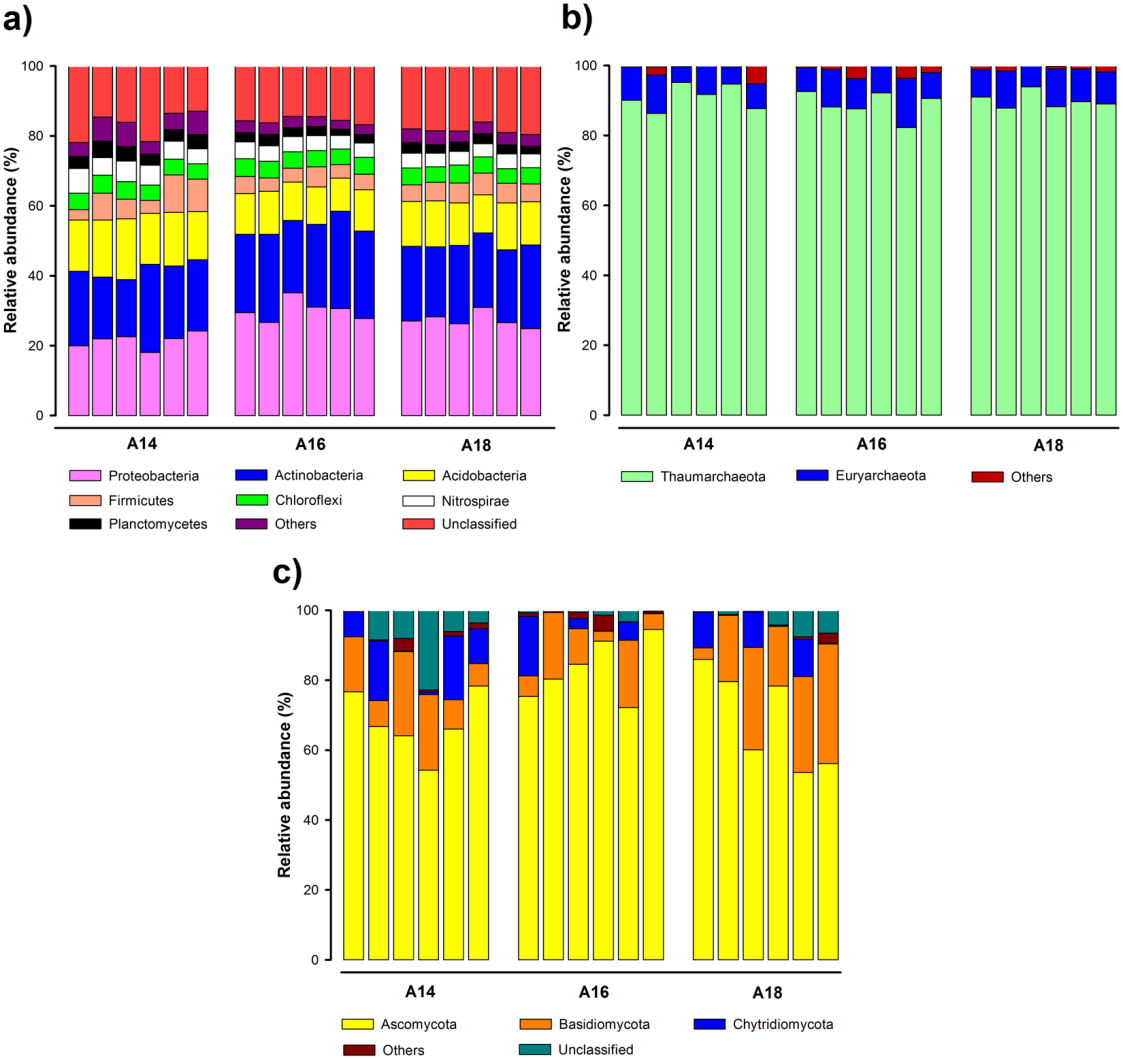
Relative abundance of bacterial (**a**), archaeal (**b**) and fungal (**c**) phyla found in the studied layers A14 (upper), A16 (intermediate) and A18 (lower).

Illumina analysis of bacterial community in soil samples of the three layers generated a total of 537,688 high quality sequences, distributed among 4,180 OTUs (operational taxonomic unit). These sequences were classified into 27 different phyla, being the predominant ones: *Proteobacteria* (the number of classified sequences in this phylum ranged from 18.0 to 35.1% in the 18 libraries), *Actinobacteria* (16.3–27.8%), *Acidobacteria* (9.5–17.4%), *Firmicutes* (3.0–10.7%) and *Chloroflexi* (4.2–5.2%); these five phyla accounted for more than 63% of total sequences (Fig. 2a). *Gammaproteobacteria*, *Alphaproteobacteria* and *Betaproteobacteria* classes predominated among *Proteobacteria*; *Actinomycetales* and *Gaiellales* orders among *Actinobacteria*; Gp6, Gp7 and Gp4 subgroups among *Acidobacteria*; and *Clostridia* and *Bacilli* classes among *Firmicutes*.

Archaeal community analysis yielded a total of 60,046 archaeal sequences, which were distributed among 101 OTUs. These reads were classified among six different phyla; although the two most dominant phyla, *Thaumarchaeota* (the number of classified sequences in this phylum ranged from 82.3 to 95.2% in all the libraries) and *Euryarchaeota* (4.5–14.1%) accounted for more than 94% of the total number of sequences across all the analyzed libraries (Fig. 2b). Other phyla such as *Pacearchaeota*, *Aenigmarchaeota*, *Woesearchaeota* and *Crenarchaeota* were also detected. *Nitrososphaerales* and *Nitrosopumilales* classes dominated among *Thaumarchaeota*, while *Thermoplasmata* class predominated among *Euryarchaeota*.

Regarding the fungal community, 1,589,549 sequences were obtained across the 18 libraries, which were distributed among 260 different OTUs. Taxonomic affiliation of the reads showed that *Ascomycota* (the number of classified sequences in this phylum ranged from 53.6 to 94.5% in all the libraries), *Basidiomycota* (2.8–34.2%) and *Chytridiomycota* (0.2–18.2%) were the predominant phyla, which accounted for more than 88% of the total number of sequences (Fig. 2c). *Zygomycota* and *Glomeromycota* phyla were also detected, but in a very low proportion. *Sordariomycetes*, *Dothideomycetes* and *Eurotiomycetes* classes dominated among *Ascomycota*, while *Agaricomycetes* and *Microbotryomycetes* classes predominated among *Basidiomycota*.

### Diversity and structure of microbial communities

#### Bacterial community

**Figure 3:**
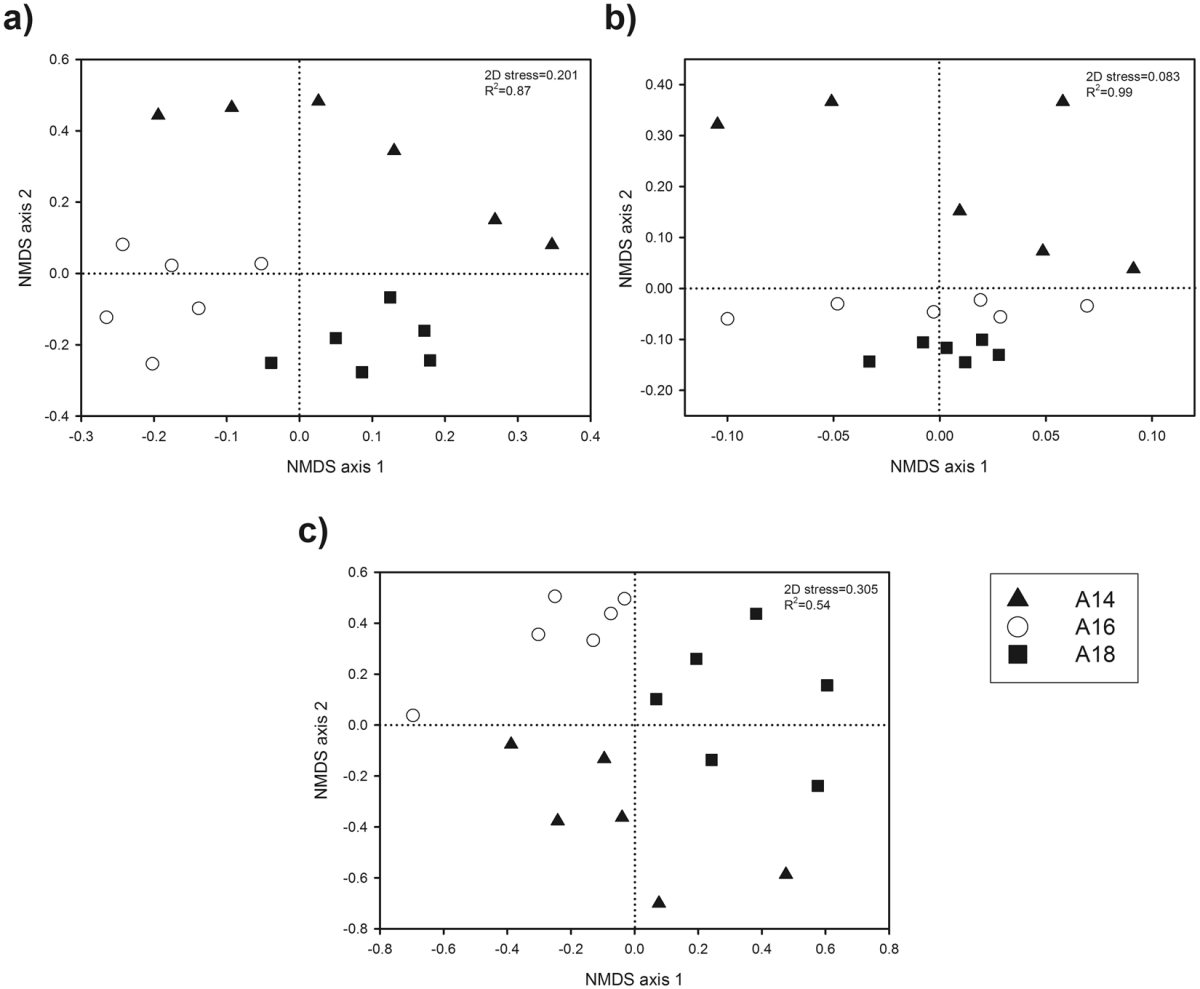
Non-metric multidimensional scaling (NMDS) ordinations based on Bray-Curtis similarities of OTU-based bacterial (**a**), archaeal (**b**) and fungal (**c**) community structures found in the layers A14 (upper), A16 (intermediate) and A18 (lower).

**Figure 4:**
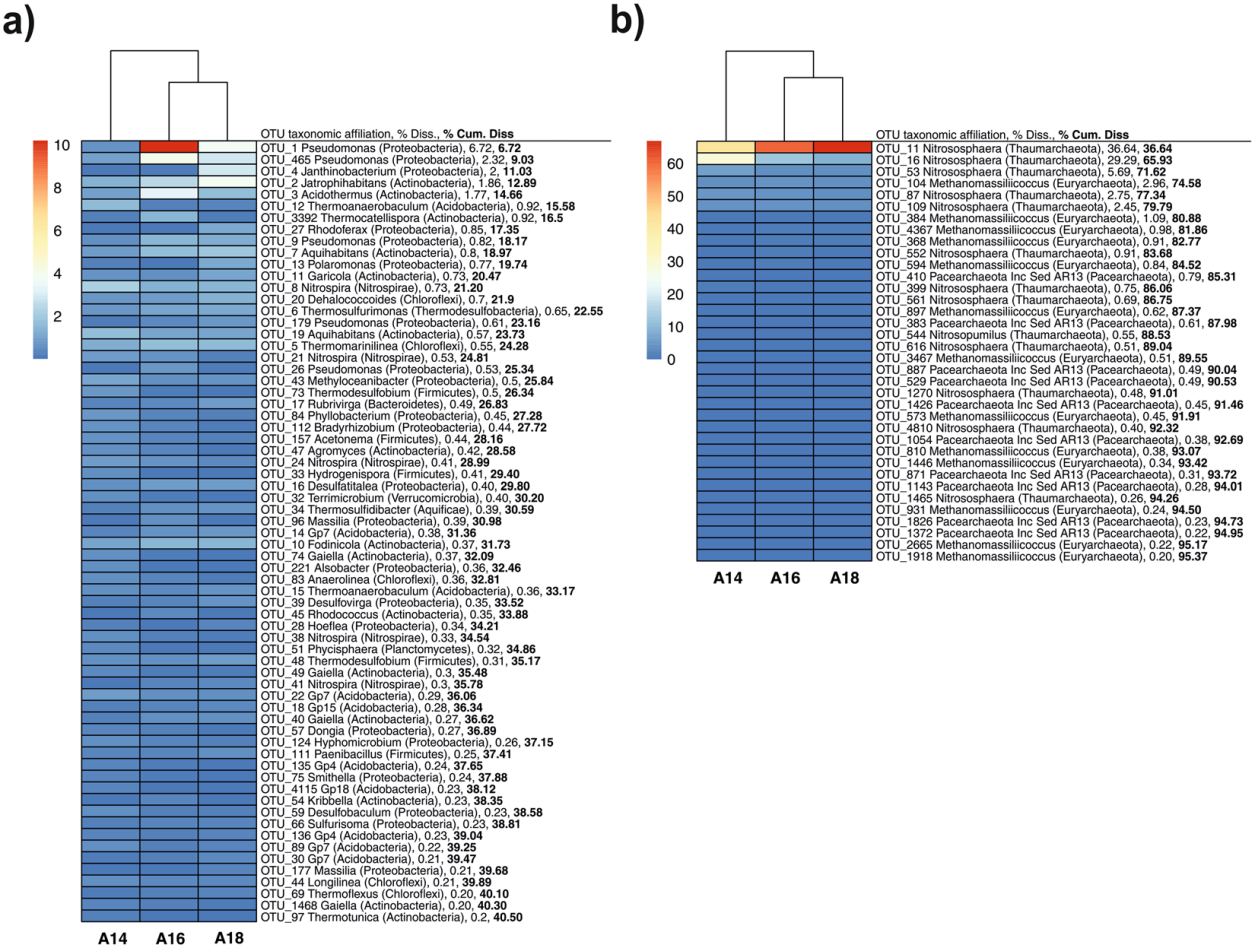
Heat map showing the relative abundance of top bacterial (**a**) and archaeal (**b**) OTUs contributing to differences (SIMPER analysis) in community structures among the studied layers A14 (upper), A16 (intermediate) and A18 (lower). Only OTUs contributing ≥0.2% of dissimilarity among layers were selected for heat map representation and they were represented in decreased order of dissimilarity contribution. Sites were clustered using UPGMA dendrogram based on Canberra distances. Percentages to the right of each OTU indicate the percent dissimilarity contributed by each OTU (% Diss.) and the cumulative percent dissimilarity among layers (% Cum. Diss.). Color legend and scale (%) are provided in the figure.

Bacterial richness, Chao 1 and ACE richness estimators as well as evenness did not significantly vary among the soil samples of the three superimposed layers. Instead, Shannon index was shown to increase in the upper layer A14 respect to the other two layers (Table S1). According to global and pairwise PERMANOVA and ANOSIM, bacterial community structures significantly differed among the three layers (Table 2), which was corroborated by NMDS (non-metric multidimensional scaling) ordination since samples were mainly grouped according to the layers they were sampled from (Fig. 3a). However, SIMPER analysis showed that dissimilarity between the bacterial community composition of the intermediate and lower layers A16 and A18 (36.9%) was lower than that of the upper layer A14 and the intermediate (A16; 52.5%) and lower layer (A18; 52.9%). The dissimilarity in bacterial community composition among the three layers was clearly noticed at taxonomic level. At class level, *Alphaproteobacteria*, *Nitrospira*, Gp6 and Gp4 subgroups, *Clostridia* and *Planctomycetia* were significantly more abundant in the upper layer A14; *Actinobacteria* (mainly *Actinobacteridae* subclass) and *Gammaproteobacteria* dominated in the intermediate layer A16; and *Betaproteobacteria* and *Anaerolineae* (with no differences respect to A16) were found to a higher extent in the lower layer A18 (Table S3). At OTU level, SIMPER analysis evidenced that the top 67 bacterial OTUs only comprised 40.5% of the dissimilarity among the three layers (Fig. 4a). Overall, 48 OTUs significantly varied among the three layers; among them, those successfully classified at genus level (considering a confidence threshold >50%) as *Pseudomonas*, *Rhodoferax*, *Polaromonas*, *Nitrospira* and *Thermodesulfobium*, contributed the most to community composition dissimilarity among the three layers (Table S4).

#### Archaeal community

In terms of richness and diversity, archaeal community did not vary among soil samples of the three layers (Table S1). Only significantly higher evenness values were detected in the upper layer A14. Regarding the community structure, although PERMANOVA and ANOSIM demonstrated that OTU-based structure of archaeal community significantly differed among the three layers and between each pair of layers (Table 2), NMDS ordination clustered together the samples of the intermediate and lower layers A16 and A18 over the NMDS axis 2, evidencing a higher similarity of archaeal community composition between these two layers in comparison with the upper layer A14 (Fig. 3b). This finding was corroborated by SIMPER pairwise comparisons, which showed that dissimilarity of archaeal community composition between layers A16 and A18 (10.9%) was lower than that between layers A14 and A16 (28.1%) as well as between layers A14 and A18 (30.8%). The top 36 OTUs reported from SIMPER analysis contributed to 95.4% of the total dissimilarity and represented 98.5% of the normalized number of sequences (Fig. 4b). Although no significant differences in the relative abundance of the different phyla and classes were detected among the three layers (Table S5), some OTUs (OTU11, OTU16, OTU552, OTU561 and OTU1270) classified as belonging to *Nitrososphaera* genus significantly differed among the three layers (Table S6).

#### Fungal community

**Figure 5:**
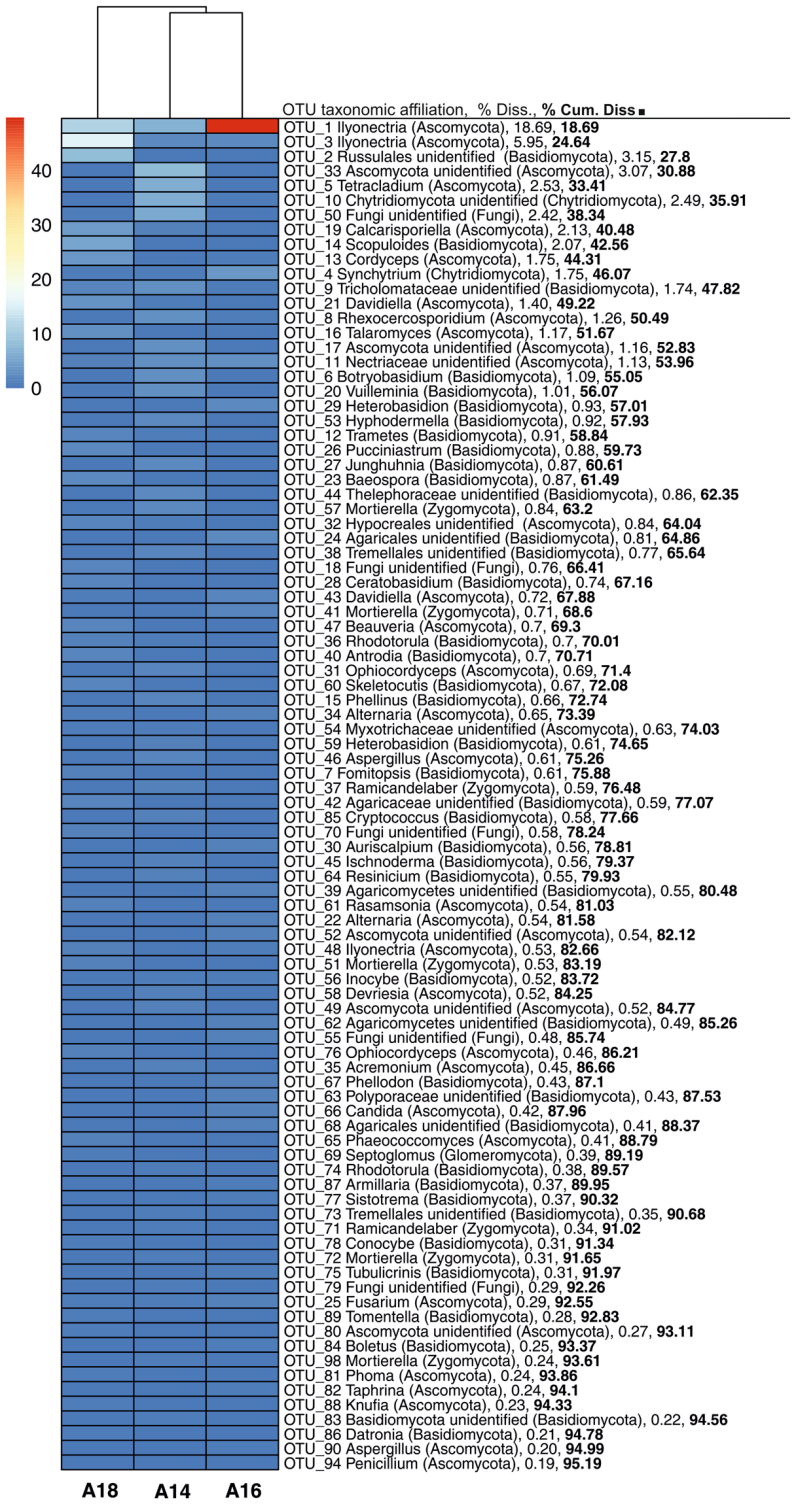
Heat map showing the relative abundance of top fungal OTUs contributing to differences (SIMPER analysis) in fungal community structures among the studied layers A14 (upper), A16 (intermediate) and A18 (lower). Only OTUs contributing ≥0.2% of dissimilarity among layers were selected for heat map representation and they were represented in decreased order of dissimilarity contribution. Sites were clustered using UPGMA dendrogram based on Canberra distances. Percentages to the right of each OTU indicate the percent dissimilarity contributed by each OTU (% Diss.) and the cumulative percent dissimilarity among layers (% Cum. Diss.). Color legend and scale (%) are provided in the figure.

Fungal communities in soil samples of the three layers did not significantly vary in terms of richness, diversity or evenness (Table S1). Instead, global and pairwise PERMANOVA and ANOSIM demonstrated significant differences of OTU-based structure of fungal community (Table 2). These results were corroborated by NMDS ordination, which clustered the samples in three different groups according to the layer they belonged to (Fig. 3c). SIMPER analysis showed that dissimilarity degree of the fungal community structures between each pair of layers was comparable (A14 *vs*. A16 = 84.1%; A14 *vs*. A18 = 87.2%; A16 *vs*. A18 = 81.6%). The differences in soil fungal communities among the layers were also evidenced at taxonomic level; *Sordariomycetes* class was significantly more abundant in the intermediate layer A16, while *Leotiomycetes* predominated in the upper layer A14 (Table S7). At OTU level, SIMPER analysis showed that top 82 OTUs contributed to 95.2% of the total dissimilarity among the three layers (Fig. 5). OTUs classified as *Tetracladium* (OTU5), unidentified *Ascomycota* (OTU17 and OTU49) and *Ramicandelaber* predominated in the upper layer A14, other OTUs belonging to *Ilyonectria* (OTU1) and *Davidiella* (OTU43) genera were found to a higher extent in the intermediate layer A16, and OTUs classified as *Ilyonectria* (OTU3) or unidentified *Russulales* (OTU33) were significantly more abundant in the lower layer A18 (Table S8).

## Discussion

Changing features of microbial communities in human impacted soils of ancient settlements can be used as a tool to evidence different anthropogenic activities in the distant past^5,8,10,11^. In this study, soil bacterial, archaeal and fungal communities in three layers, excavated at the archaeological site on Monte Iato and believed to have been created in a chronological order in the context of cultic feasts during the 6^th^ cent. BC, were investigated in terms of abundance, physiological profile, diversity and community structure. To the best of our knowledge, this is the first description of diversity of all three microbial domains inhabiting an archaeological site at the same time. In a previous work, bacterial and fungal communities associated with soil samples collected from a ritual deposit (food waste disposal) in the main room of an indigenous building and above the fireplace in the annex of the same building, from the same archaeological area (Monte Iato settlement) as studied here, were characterized in terms of abundance and taxonomic composition^5^. Since, on one hand, archaeological investigations proved clearly that the ancient human activities responsible for soil formation at the locations studied in both works differed completely^17–20^, and, on the other hand, environmental conditions at the studied sites were shown to be similar (as demonstrated by the non-significant differences in soil physicochemical properties between samples from both studies (data not shown)), the differences in the features of soil microbial communities inhabiting locations from both surveys are thus mainly driven by the impact of the different past human activities. In this way, samples analyzed in our earlier survey were used as reference for the samples investigated in this study to discuss the results obtained for bacterial and fungal communities, as shown below.

Soil samples of the three studied layers were characterized by low C and N contents. These low nutrient contents, along with other environmental factors presumably present in buried layers, such as low moisture, scarce oxygen availability and poor gaseous exchange, were probably restricting microbial life. However, our analyses demonstrated, in all soil samples of the three layers, the presence of both total (analyzed through DNA-based qPCR analyses) and living biomass (analyzed through PLFA analyses, which target only living microorganisms)^23^. Unfortunately, there are no previous studies quantifying the total abundance of microbial communities at archaeological sites in order to do comparisons in absolute terms. The upper layer A14 showed significantly increased levels of organic C, which were not concomitant with an increment in living bacterial or fungal biomass, probably because this C increase is insufficient to sustain higher microbial biomass. Also Peters, *et al*.^11^, studying microbial communities in different anthropogenically-impacted soils of a Late Bronze Age settlement located in the North Caucasus (Russia), did not find a positive correlation between higher organic C contents and increased levels of microbial biomass (as determined by DAPI (4′, 6-diamino-2-fenilindol) staining). Yet, we observed significantly increased copy numbers of *gtlA* gene in soil samples of the layer A14 respect to soil samples of the other two layers, which could indicate higher bacterial respiration rates in this layer due to the presence of a higher amount of easily decomposable C sources. CLPP assays showed that microbial C-consumption patterns differed among soil samples of the three layers; easily decomposable carbohydrates such as pyruvic acid methyl ester and D-xylose were consumed to a higher extent in the upper layer A14, while more recalcitrant C compounds such as Tween 80 and Tween 40 were preferably consumed in deeper layers A16 and A18. We thus see the results of CLPP analysis in connection with the potential higher bacterial respiration detected in upper layer A14. Interestingly, also Peters, *et al*.^11^, in the aforementioned study, found higher microbial respiration rates in soils with higher organic C content.

The impact of human activities on soil samples of the three layers studied here seems clear since they were created in a chronological order on top of each other in order to produce a compact surface for the erection of ephemeral buildings or tents in the context of periodic cultic feasts^21^. The specific human activities as well as the characteristics of the remains left by humans (whose presence was evidenced by the artifacts found, e.g., ceramics, ash remains, charcoal, animal bone fragments, etc.) likely differed among the three layers, which presumably determined the heterogeneity of the original physicochemical and environmental conditions (influencing the characteristics of original soil microbial communities) existing in the three layers. These conditions thus converted these three layers into a suitable system to assess whether microbial communities can act as ecofacts (bioindicators) of human activities in the distant past. In concordance with our hypothesis, we found significant differences in the structure of the soil microbial communities among the three layers; i.e., each layer presented a specific bacterial, archaeal and fungal community structure. Previous studies have argued that present microbial communities existing in an archaeological human-impacted soil are the same as those originally inhabiting the soil^11^. However, although soil bacterial and archaeal community structures differed between the intermediate and lower layers A16 and A18, the higher similarity among them in comparison with the upper layer A14 may be the result of a similar adaptation to changing environmental conditions over the time. Since we analyzed soil microbial communities using DNA-based methods, both living and non-living (i.e., dead or in a dormant state) microorganisms were targeted^23^, capturing thus the soil microbial dynamics in the three layers over the time.

The three studied layers harboured a huge microbial diversity in taxonomic terms. In the case of bacterial community, 27 different phyla (using a 50% confidence threshold for taxonomic affiliation of sequences) were shown to inhabit soils of these layers. Xu, *et al*.^6^ also found an unexpectedly high Illumina-based bacterial diversity in buried soil samples at the No. 1 Wangshanqiao Chu tomb in China. The community structure of the bacterial community in the studied layers was significantly different (PERMANOVA, F = 7.961, *p* = 0.0001; ANOSIM, r = 0.759, *p* = 0.0001) from that reported in our previous study on soils (of a ritual deposit (A6 and A7) and above the fireplace of a residential building (A2 and A3) of the aforementioned protohistorical dwelling^19,20^) at the same archaeological area on Monte Iato as studied here^5^. Pairwise PERMANOVA and ANOSIM also showed that structure of bacterial communities significantly differed between each pair of archaeological locations from both studies (Table S9). At taxonomic level, we found in the soils of the residential building a significantly higher abundance of *Actinobacteria* and *Planctomycetes*, while *Proteobacteria*, *Firmicutes*, *Chloroflexi* and *Nitrospirae* were detected to a significantly higher extent in soils of the present study (Table S10). This finding points to the high variability of the composition of soil bacterial communities within an ancient settlement as a consequence of the impact of different ancient human activities. Our data, along with those of other surveys of soil bacterial communities at archaeological sites using high-throughput sequencing techniques^6^, demonstrate that the taxonomic composition of soil bacterial communities concurs with that described as common in the soil environment^24,25^ and evidence that bacterial diversity in these environments is not dominated by “rare” microbial taxa. However, archaeological sites have been described as potential sources of novel microbial species^5,9^. In the present study, OTUs affiliated to genus *Pseudomonas* were shown to be the most abundant ones and the main responsible for dissimilarity in soil bacterial community structure among the three layers. This genus is widely distributed in soil environment and many *Pseudomonas* species are able to adapt to variable nutritional (both oligotrophic and copiotrophic) environments and pH conditions^26,27^. Previous studies have confirmed the presence of *Pseudomonas* in archaeological remains. In a culture-dependent study of the mud from a Roman thermae, strains classified as *Pseudomonas* were reported^9^. Xu, *et al*.^6^ suggested that *Pseudomonas*, among other bacterial genera, could play a key role in degrading organic culture heritage in burial conditions.

In comparison with bacterial community, soil archaeal community showed a much more limited diversity in the studied layers as it was basically comprised of *Thaumarchaeota* and *Euryarchaeota* phyla, which have been shown to dominate soil habitats^28^. OTUs affiliated to *Nitrososphaera* genus (*Thaumarchaeota*) were the most abundant and contributed the most to dissimilarity of community structures among the three layers. The variations in the OTUs belonging to *Nitrososphaera* are probably a consequence of qualitative and quantitative changes in ammonia and carbon sources among the three layers over time^29^.

Soil fungal community inhabiting the studied layers was also highly diverse and its structure significantly differed (PERMANOVA, F = 3.225, *p* = 0.0001; ANOSIM, r = 0.496, *p* = 0.0001; Table S9) from that of the aforementioned soils at the ritual deposit and fireplace sites (i.e., differently impacted by ancient human activities)^5^. These differences in community structure were evidenced at taxonomic level, while *Dothideomycetes* and *Pezizomycetes* classes were significantly more abundant in the soils of the protohistorical dwelling, *Sordariomycetes* and *Agaricomycetes* were significantly more dominant in the soils of the present study (Table S11). All of these fungi are commonly found in soil environments^30^. As for bacteria, these findings evidence the changing taxonomic composition of fungal communities at ancient settlements as a consequence of anthropogenic activities. Previous microbial studies of human settlements prove that fungal communities provide useful information regarding human activities in the past. For example, the accumulation of keratinophilic fungi at specific sites of a settlement has been considered as a proof of the use of those sites as domestic animal shelters, household pits or larders of wool, skins and feathers. The detection of phytopathogenic fungi such as *Fusarium* spp. at a cultural site of a medieval settlement has been associated with grain pit locations^10^. In this line, in our study, the presence of OTUs belonging to *Ilyonectria* genus (which were the most abundant ones and contributed the most to explain the differences in the fungal community structures among the three layers) in soil samples of the three layers is seen as an evidence of the accumulation of vegetal material (whose presence was also revealed by the charcoal detected) as a consequence of the ancient anthropogenic activity since species of this genus have been associated with root and decay of woody and herbaceous plants^31,32^.

In conclusion, we found representatives of all three microbial domains (bacteria, archaea and fungi) in the soil samples of all three studied layers. Soil bacterial, archaeal and fungal communities were characterized by a wide diversity, which confirms our first hypothesis. We also demonstrated that soil microbial communities of the three layers differed significantly (in agreement with our second hypothesis) in terms of C-source consumption patterns, community structure and taxonomic composition and relate this fact to the different past human activities that resulted in heterogeneous conditions for soil microbial communities in each of the layers. The high degree of similarity between microbial characteristics (C-utilization patterns and the structure of bacterial and archaeal communities) in the intermediate and lower layer and the dissimilarity of these two layers with the upper layer might be a consequence of the adaptation of microbial communities to the prevailing physicochemical and environmental conditions in each layer. Soil bacterial and fungal communities studied here significantly differed from those in other human-impacted soils of the same settlement, which demonstrates the high variability of microbial communities within an ancient settlement. Altogether, our data evidence that changing characteristics of soil microbial communities reflect different ancient human activities. In the future, further works studying the features of soil microbial communities from a wider range of archaeological locations are needed to identify key microbial groups as indicators of specific past anthropogenic activities and to generate more general conclusions regarding the use of the characterization of soil microbial communities as a complementary tool of the traditional archaeological methods.

## Methods

### Site description and soil sampling

**Figure 6:**
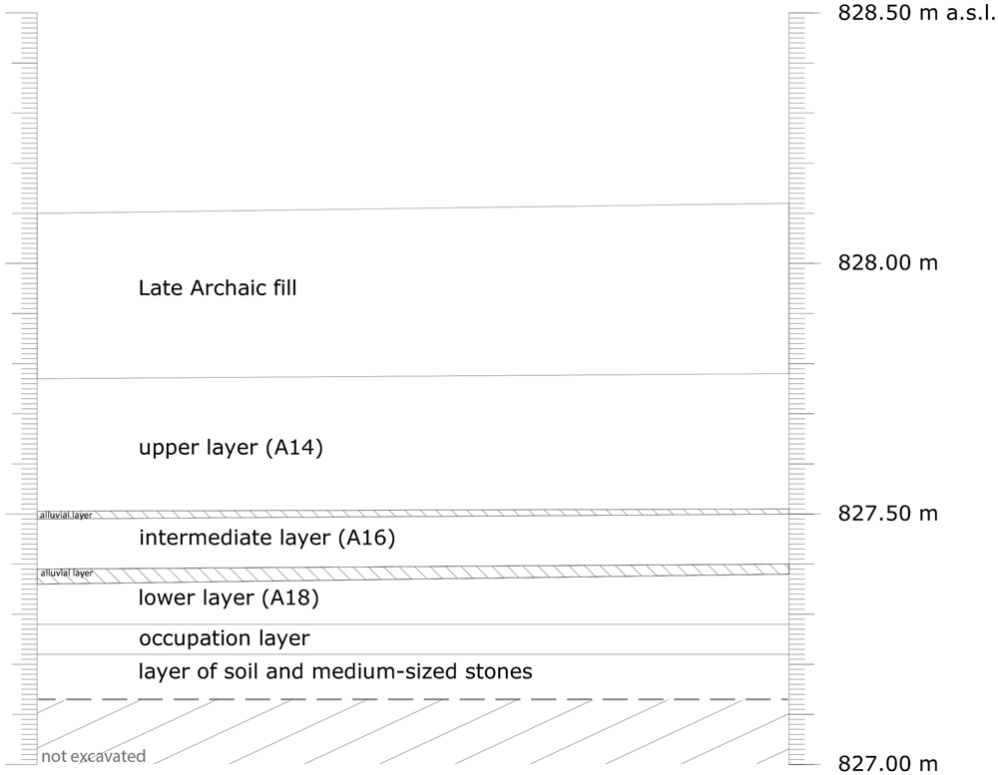
Schematic representation of the excavated area at the archaeological Monte Iato settlement showing the studied layers A14 (upper), A16 (intermediate) and A18 (lower).

The three clearly separated layers described in the introduction were discovered during a field campaign in September 2015. An area of 1.5 × 1.5 m, with a depth of 50 cm, was excavated. The superimposed layers of stone packings were clearly separated by thin layers (a few cm) of alluvial material The upper layer (A14) had a depth of 27 cm while the intermediate (A16) and lower layers (A18) measured 10 cm each (Fig. 6). The upper layer was covered by a late archaic 35 cm thick fill for the construction of a ramp around 500 BC. Below the lower layer an occupation layer as well as another layer of soil and medium-sized stones came to light.

For each of the three layers (A18, A16, A14), three sampling points in the vertical mid of each layer were chosen, which were separated by ca. 40 cm from each other. From each of these sampling points, several sub-samples were collected with sterile spatulas, trying to get as much soil as possible and avoiding to sample stones or remains, merged and placed in sterile vials. This way, we obtained 3 soil samples (biological replicates) per layer. Immediately after sampling, the soil samples were stored in cooled boxes, shipped to the laboratory in Innsbruck (Austria) and stored at 4 °C for physicochemical characterization and measurement of microbial activities or at −80 °C prior to DNA-based and PLFA analyses.

Unfortunately, there is no information available in the literature on soil microbial communities at places in the vicinity of the studied archaeological site. Therefore, we could not include a pristine reference site in our studies.

### Physicochemical characterization of samples

Each of the 9 soil samples was characterized regarding pH (CaCl_2_), dry mass (105 °C for 24 h) and contents of soil organic matter (SOM; mass loss on ignition at 600 °C), total organic carbon (TOC; calculated from SOM) and total N (CHN Analyzer). C/N ratio was calculated as TOC/N.

### Community level physiological profiles

The Biolog EcoPlate system (BIOLOG Inc., CA, USA) was used to determine community level physiological profiles (CLPP) for each soil sample in triplicate according to Siles, *et al*.^33^. Briefly, 1 g of soil was shaken in 10 ml of sterile saline solution (0.85% w/v NaCl) during 1 h and soil suspensions were then serially diluted to 10^−4^. 130 µl of soil solutions were used for each well and Ecoplates were incubated at 20 °C for 21 days. Colour development for each well was obtained in terms of optical density (OD) at 590 nm every 24 h using an automated plate reader. Microbial catabolic activity was calculated as average well colour development (AWCD) as described by Siles, *et al*.^33^. The 10-day OD data, normalized by dividing them by their AWCD, were selected to calculate substrate richness, Shannon index, evenness and the relative consumption rate of each substrate.

### Analysis of phospholipid fatty acids

Phospholipid fatty acid (PLFA) analysis was used as a proxy to quantify living microbial biomass in each soil sample. Extraction and analysis of PLFAs were carried out as previously described^34^. Briefly, microbial lipids were firstly extracted from 0.5 g of soil sample using a mixture of chloroform:methanol:phosphate buffer (1:2:0.8; v/v/v) according to Bligh and Dyer^35^. Phospholipids were then separated by solid-phase extraction cartridges (LiChrolut Si 60, Merck), and the samples were subjected to mild alkaline methanolysis. The free methyl esters of phospholipid fatty acids were analyzed by gas chromatography-mass spectrometry (GC-MS; 450-GC, 240-MS ion trap detector, Varian, Walnut Creek, CA).

The PLFA i14:0, i15:0, a15:0, 16:1ω9, 16:1ω7, 16:1ω5, 10Me-16:0, i17:0, a17:0, cy17:0, 17:0, 10Me-17:0, 10Me-18:0 and cy19:0 were addressed as bacterial signature markers. The PLFA 10Me-16:0, 10Me-17:0 and 10Me-18:0 were used as actinobacterial markers, while the fatty acid 18:2ω6,9 was selected as fungal marker. The fatty acids found both in bacteria and fungi, such as 15:0 and 18:1ω7, were excluded from the analysis characterizing the different microbial groups^36^. The sum of all the identified phospholipids was used to estimate the total microbial biomass. Ratios between PLFA markers derived from fungi and bacteria (F/B) were calculated.

### DNA extraction

Total DNA from each of the 9 soil samples was extracted in triplicate from 250 mg of soil fresh mass, using Power Soil™ DNA Isolation Kit (MO BIO Laboratories Inc., Solana Beach, USA). Triplicate DNA extracts from each soil sample were then pooled, and DNAs were purified using DNA Clean & Concentrator Kit (Zymo Research, Irvine, USA) according to manufacturer’s instructions. The quality of DNAs was spectrophotometrically checked by NanoDrop (Thermo Fisher Scientific Inc., Bremen, Germany) based on the absorbance ratios A_260_/A_280_ and A_260_/A_230_. Subsequently, the DNAs were quantified using QuantiFluor™ dsDNA System (Promega, Madison, USA) and DNA concentration for each sample was standardized to 1 ng μL^−1^.

### Quantitative PCR analyses

Quantitative PCR (qPCR) was used to determine total (living and non-living (dead or in a dormant state)) bacterial, archaeal and fungal abundance and to estimate the potential aerobic bacterial respiration in each soil sample. The quantification of bacterial and archaeal 16S rRNA and fungal 18S rRNA genes as well as bacterial citrate synthase gene (*gtlA*, as functional marker of bacterial respiration activity) was carried from each of the 9 DNA samples in duplicate with the pairs of primers Eub338/Eub518 for bacteria^37^, Arch-967F/Arch-1060R for archaea^38^, FR1/FF390 for fungi^39^ and CS680F/CS904R for *gtlA* gene^40^ using SYBR^®^ Green as detection system (Bio-Rad, Hercules, USA). The PCR mixtures and thermal conditions for quantification of ribosomal bacterial, archaeal and fungal genes were as previously described^41^. In the case of *gtlA* gene, the amplifications were carried out using the PCR mixtures described by Siles and Margesin^41^ under the following thermal conditions: 94 °C for 5 min followed by 40 cycles of 94 °C for 20 s, 61 °C for 30 s, and 72 °C for 30 s. In all the cases, after amplification reactions, melting curve and gel electrophoresis analyses were conducted to confirm that the amplified products had the appropriate size. Copy number for each gene was quantified by comparing the cycle at which fluorescence crossed a threshold to a standard curve constructed using a serial dilution of a plasmid containing an appropriate template, as previously described^41^. Standard curve for *gltA* gene was generated using *Sphingomonas alpina* S8-3^T^ (DSM 22537) genomic DNA. The relations A/B (archaeal 16S rRNA gene copy number/bacterial 16S rRNA gene copy number) and F/B (fungal 18S rRNA gene copy number/bacterial 16S rRNA gene copy number) were calculated on the basis of the log-transformed copy number values.

### Bacterial and archaeal 16S rRNA gene fragment and fungal internal transcribed spacer 1 sequencing

For Illumina amplicon sequencing analyses, two technical replicates of each of the 9 DNA samples were carried out, obtaining a total of 18 libraries for each microbial (bacterial, archaeal and fungal) community. Diversity and structure of bacterial communities were characterized by amplifying a fragment of 16S rRNA gene (and capturing the hypervariable V1-V3 regions) with the primers 27 F (5′-AGRGTTTGATCMTGGCTCAG-3′) and 519 R (5′-GTNTTACNGCGGCKGCTG-3′)^42^. For each library, DNA samples were amplified in triplicate using the PCR mixtures and thermal conditions previously described^5^. The success of the amplification was checked in a 2% agarose gel and reactions of the same library were merged. Subsequently, PCR products from all the libraries were purified using Agencourt AMPure XP magnetic beads kit (Beckman Coulter, Inc., Pasadena, USA), quantified with the QuantiFluor™ dsDNA System and pooled in equal proportions. The pooled product was then used to prepare Illumina DNA library following TruSeq DNA library preparation protocol. Paired-end sequencing (2 × 300) was performed on the Illumina MiSeq sequencing platform (Illumina, San Diego, USA) at MR DNA (www.mrdnalab.com, Shallowater, TX, USA).

Archaeal communities were analyzed using the pair of primers arch519F (5′- CAGCMGCCGCGGTAA-3′) and arch806R (5′-GGACTACVSGGGTATCTAAT-3′), which captures the V4 hypervariable region of 16S rRNA gene. DNA samples were amplified in triplicate using HotStarTaq *Plus* Master Mix Kit (Qiagen, Valencia, USA) and barcoded forward primers, under the following thermal conditions: initial denaturation at 94 °C for 3 minutes, followed by 28 cycles of denaturation at 94 °C for 30 s, primer annealing at 50 °C for 40 s and extension at 72 °C for 60 s as well as a final elongation step at 72 °C for 5 minutes. The processing of PCR products for paired-end sequencing (2 × 300) on Illumina MiSeq sequencing platform was done as described for bacteria.

In the case of fungal communities, internal transcribed spacer (ITS) 1 region was firstly amplified using the ITS1F (5′-CTTGGTCATTTAGAGGAAGTAA-3′) and ITS2 (5′-GCTGCGTTCTTCATCGATGC-3′) primers^43,44^ under the PCR and thermal conditions reported by Margesin, *et al*.^5^. Secondly, the processing of PCR products for paired-end sequencing (2 × 300) on Illumina MiSeq sequencing platform was conducted as described for bacteria.

The raw bacterial, archaeal and fungal sequences associated with this study were deposited in the GenBank SRA database under BioProject accession number PRJNA393946.

### Processing of Illumina sequencing data

First, raw MiSeq paired-end reads from bacterial and archaeal 16S rRNA and fungal ITS1 amplicons were independently assembled and reoriented using MR DNA pipeline. Subsequently, sequences were demultiplexed and formatted for processing using a Phython script^41^. Next, sequences from each library were processed using USEARCH pipeline and UPARSE-OTU algorithm^45^. Briefly, reads were separately quality-filtered (allowing a maximum e-value of 1), trimmed (to 450-bp (base pair), 220-bp and 230-bp for bacterial, archaeal and fungal libraries, respectively), dereplicated and sorted by abundance (removing singleton sequences) prior chimera detection and OTU (operational taxonomical unit) determination at 97% sequence identity. Finally, original high-quality sequences were mapped to OTUs at the 97% identity threshold obtaining three OTU tables, one for each microbial community. The taxonomic affiliation of each OTU was obtained using RDP (ribosomal database project) taxonomic classifier^46^ against 16S rRNA training set 16 for bacterial and archaeal sequences and UNITE Fungal ITS train set 07-04-2014 for fungal sequences with a 50% confidence threshold for the three communities. Next, OTUs classified as Bacteria were removed from archaeal OTU table, and *vice versa*. In the case of archaeal dataset, also the OTUs that could not be classified as Archaea were discarded. All the remaining bacterial OTUs and all the OTUs from fungal dataset could be classified as Bacteria and Fungi, respectively, and they were thus retained. These three OTU tables were used for downstream analyses.

For diversity characterization of microbial communities and statistical analyses, the number of sequences per sample was normalized to 16,000, 1,399 and 12,000 for bacterial, archaeal and fungal OTU-tables, respectively. The bacterial, archaeal and fungal communities were characterized in terms of diversity by calculating richness (number of OTUs), Shannon index, Smith-Wilson evenness and the richness estimator indices ACE (abundance-based coverage estimation) and Chao 1 using Mothur v.1.39.3^47^.

### Statistical analyses

One-way analysis of variance (ANOVA) was applied to determine significant differences (p ≤ 0.05) among the three layers regarding: (i) soil physicochemical properties; (ii) PLFA- and qPCR-based measurements of microbial abundances; (iii) diversity measurements of CLPP dataset as well as bacterial, archaeal and fungal communities; (iv) relative abundance of bacterial, archaeal and fungal taxonomic groups; and (v) relative abundance of the top bacterial, archaeal and fungal OTUs extracted from SIMPER analysis. When ANOVA resulted in significant results, Tukey’s honest significance difference (HSD) post-hoc test was subsequently applied for multiple comparisons of means at a 95% confidence interval. Normality and heteroscedasticity of data were tested by the Shapiro-Wilk and Levene tests, respectively. In case that one of those conditions was not met, the values were log transformed.

Multivariate ordination methods were applied to reduce the multiple dimensions in datasets and visualize patterns. CLPP dataset was subjected to principal component analysis (PCA) since a previous detrended correspondence analysis of the data revealed a gradient length of the first axis <1. In the case of bacterial, archaeal and fungal community structures, non-metric multidimensional scaling (NMDS) analysis based on Bray-Curtis similarities at OTU level was used as ordination method. The significance of the differences found in CLPPs and OTU-based structures of bacterial, archaeal and fungal communities among the layers was assessed through permutational multivariate analysis of variance (PERMANOVA) and analysis of similarity (ANOSIM) with Bray-Curtis similarities after 9,999 permutations using the “vegan” package in R. PERMANOVA and ANOSIM were also used to compare the structure of soil bacterial and fungal communities of the present study with those of our previous survey investigating other soil samples of the same archaeological settlement^5^. SIMPER (similarity percentage) analysis was applied to assess which C sources and microbial OTUs were primarily responsible for the observed differences using PAST ver. 3.07 software. One heat map for each microbial community, considering the OTUs contributing ≥0.2% to microbial community dissimilarities among the layers, was modeled with the “pheatmap” package in R.

## Electronic supplementary material

Supplementary Material
