# Supplementary material for: Characterization of soil bacterial, archaeal and fungal communities inhabiting archaeological human-impacted layers at Monte Iato settlement (Sicily, Italy)
Source: Sci Rep. 2018 Jan 30;8:1903. doi: 10.1038/s41598-018-20347-8 (PMC5789874; doi:10.1038/s41598-018-20347-8)
Supplement: Supplementary file 1 — Supplementary Material [file 41598_2018_20347_MOESM1_ESM.pdf]

SUPPLEMENTARY MATERIAL

Characterization of soil bacterial, archaeal and fungal communities inhabiting archaeological human-impacted layers at Monte Iato settlement (Sicily, Italy)

José A. Siles, Birgit Öhlinger, Tomas Cajthaml, Erich Kistler, Rosa Margesin*

*Corresponding author: Rosa Margesin; E-mail address: rosa.margesin@uibk.ac.at

Scientific Reports

The supplementary material includes Tables S1-S11

Table S1. Diversity characteristics of CLPPs (community level physiological profile) as well as archaeal, bacterial and fungal communities found in soil samples of the studied layers A14 (upper), A16 (intermediate) and A18 (lower). The data represent the mean values of the six replicate libraries analyzed in each layer. For each variable, data followed by different letters are significantly different according to Tukey's HSD test ($p \leq 0.05$).

	**A14**	**A16**	**A18**
**CLPP-based diversity**			
Richness	14.67 **a**	15.33 **a**	11.67 **a**
Shannon index	2.05 **a**	2.54 **b**	2.20 **ab**
Evenness	0.78 **a**	0.93 **b**	0.90 **ab**
**Bacterial community**			
Richness	1566 **a**	1418 **a**	1393 **a**
Shannon index	6.15 **b**	5.65 **a**	5.83 **a**
Chao 1	1958 **a**	1785 **a**	1743 **a**
ACE	1962 **a**	1777 **a**	1749 **a**
Evenness	0.352 **a**	0.345 **a**	0.364 **a**
**Archaeal community**			
Richness	37.17 **a**	44.83 **a**	40.67 **a**
Shannon index	1.69 **a**	1.66 **a**	1.48 **a**
Chao 1	49.94 **a**	62.12 **a**	54.50 **a**
ACE	55.57 **a**	66.83 **a**	54.82 **a**
Evenness	0.485 **b**	0.468 **ab**	0.462 **a**
**Fungal community**			
Richness	147.0 **a**	136.5 **a**	131.0 **a**
Shannon index	2.77 **a**	2.05 **a**	2.28 **a**
Chao 1	184.9 **a**	173.5 **a**	155.6 **a**
ACE	177.7 **a**	174.9 **a**	158.7 **a**
Evenness	0.502 **a**	0.498 **a**	0.494 **a**

Table S2. SIMPER (similarity percentage) analysis of CLPPs (community level physiological profile) showing the contribution of each C source to the dissimilarity among the studied layers A14 (upper), A16 (intermediate) and A18 (lower).

C-source number	C source	C-source family	Av. dissim[1]	Contrib[2] (%)	Cumulative[3] (%)	AWCD in A14[4]	AWCD in A16[4]	AWCD in A18[4]
1	L-Serine	Amino acids	6.38	10.22	10.22	0.156	0.075	0.064
2	Tween 80	Polymers	5.90	9.44	19.66	0.040	0.126	0.145
3	Tween 40	Polymers	4.92	7.87	27.54	0.001	0.124	0.093
4	L-Asparagine	Amino acids	4.07	6.52	34.06	0.023	0.087	0.120
5	L-Arginine	Amino acids	3.51	5.63	39.68	0.063	0.075	0.104
6	Pyruvic Acid Methyl Ester	Carbohydrates	3.28	5.25	44.93	0.086	0.057	0.059
7	D-Mannitol	Carbohydrates	3.04	4.86	49.80	0.023	0.083	0.067
8	Putrescine	Amines/Amides	2.72	4.36	54.15	0.036	0.050	0.056
9	Itaconic Acid	Carboxylic and Acetic Acids	2.61	4.18	58.33	0.029	0.063	0.078
10	4-Hydroxy Benzoic Acid	Carboxylic and Acetic Acids	2.43	3.90	62.23	0.005	0.050	0.057
11	N-Acetyl-D-Glucosamine	Carbohydrates	2.34	3.76	65.98	0.026	0.045	0.058
12	D-Malic Acid	Carboxylic and Acetic Acids	2.04	3.27	69.25	0.002	0.061	0.002
13	Phenylethyl-amine	Amines/Amides	2.04	3.26	72.51	0.034	0.043	0.028
14	D-Cellobiose	Carbohydrates	1.89	3.03	75.53	0.056	0.000	0.000
15	Glycyl-L-Glutamic Acid	Amino acids	1.77	2.83	78.37	0.051	0.009	0.011
16	D-Galactonic Acid/γ-Lactone	Carboxylic and Acetic Acids	1.61	2.59	80.95	0.043	0.013	0.012
17	L-Threonine	Amino acids	1.54	2.47	83.42	0.046	0.001	0.001
18	i-Erythritol	Carbohydrates	1.46	2.34	85.76	0.033	0.004	0.021
19	γ-Hydroxybutyric Acid	Carboxylic and Acetic Acids	1.45	2.32	88.08	0.039	0.003	0.010
20	Glycogen	Polymers	1.41	2.26	90.34	0.043	0.002	0.002
21	D-Galacturonic Acid	Carboxylic and Acetic Acids	1.16	1.86	92.20	0.034	0.005	0.001
22	D-Xylose	Carbohydrates	0.77	1.24	93.44	0.023	0.001	0.001
23	D-Glucosaminic Acid	Carboxylic and Acetic Acids	0.74	1.18	94.62	0.014	0.012	0.003
24	2-Hydroxy Benzoic Acid	Carboxylic and Acetic Acids	0.73	1.17	95.79	0.022	0.000	0.000
25	Glucose-1-Phosphate	Carbohydrates	0.65	1.04	96.83	0.019	0.001	0.001
26	α-Cyclodextrin	Polymers	0.59	0.94	97.77	0.017	0.000	0.000
27	L-Phenylalanine	Amino acids	0.40	0.64	98.41	0.011	0.001	0.002
28	D,L-α-Glycerol Phosphate	Carbohydrates	0.36	0.58	98.98	0.007	0.009	0.005
29	α-Ketobutyric Acid	Carboxylic and Acetic Acids	0.28	0.45	99.44	0.008	0.000	0.001
30	β-Methyl-D-Glucoside	Carbohydrates	0.28	0.45	99.88	0.008	0.000	0.001
31	α-D-Lactose	Carbohydrates	0.07	0.12	100.00	0.002	0.000	0.000

[1] Av. dissim, average dissimilarity.
[2] Contrib., contribution of each C source to group differences.
[3] Cumulative, sum of each C-source contribution to group differences.
[4] AWCD, average well colour development.

Table S3. Relative abundance (%) of the bacterial phyla and the most abundant bacterial classes found in the studied layers A14 (upper), A16 (intermediate) and A18 (lower). The data represent the mean values of the six replicate libraries analyzed in each layer. For each taxonomic group, values followed by different letters are significantly different (p≤0.05) according to Tukey's HSD test.

	**A14**	**A16**	**A18**
**Bacterial phyla**			
Proteobacteria	21.44 **a**	30.08 **b**	27.30 **b**
Actinobacteria	20.26 **a**	24.14 **b**	21.66 **ab**
Acidobacteria	15.37 **b**	11.15 **a**	12.45 **a**
Firmicutes	6.65 **a**	4.47 **a**	5.45 **a**
Chloroflexi	4.69 **a**	4.74 **a**	4.64 **a**
Nitrospirae	5.51 **b**	4.31 **a**	3.99 **a**
Planctomycetes	3.83 **a**	2.56 **a**	2.64 **a**
Bacteroidetes	1.55 **b**	0.65 **a**	0.70 **a**
Gemmatimonadetes	0.96 **ab**	0.82 **a**	1.01 **b**
Verrucomicrobia	1.44 **b**	0.58 **a**	0.45 **a**
Latescibacteria	0.57 **b**	0.31 **a**	0.45 **b**
Armatimonadetes	0.40 **a**	0.31 **a**	0.51 **a**
Others	0.52 **a**	0.33 **a**	0.43 **a**
Unclassified	16.80 **a**	15.56 **a**	18.31 **a**
**Bacterial classes**			
Actinobacteria (*Actinobacteria*)	19.86 **a**	23.75 **b**	20.95 **ab**
Gammaproteobacteria (*Proteobacteria*)	3.64 **a**	18.00 **c**	9.89 **b**
Alphaproteobacteria (*Proteobacteria*)	8.90 **b**	4.75 **a**	4.42 **a**
Betaproteobacteria (*Proteobacteria*)	3.76 **a**	3.21 **a**	8.24 **b**
Nitrospira (*Nitrospirae*)	5.51 **b**	4.31 **a**	3.99 **a**
Deltaproteobacteria (*Proteobacteria*)	4.71 **b**	3.68 **a**	4.31 **b**
Gp6 (*Acidobacteria*)	4.80 **b**	2.94 **a**	2.96 **a**
Anaerolineae (*Chloroflexi*)	3.11 **a**	3.68 **b**	3.62 **b**
Gp7 (*Acidobacteria*)	3.07 **ab**	2.76 **a**	3.56 **b**
Clostridia (*Firmicutes*)	3.71 **b**	2.22 **a**	2.86 **a**
Planctomycetia (*Planctomycetes*)	3.45 **b**	2.34 **a**	2.37 **a**
Gp4 (*Acidobacteria*)	2.58 **b**	1.53 **a**	1.59 **a**
Bacilli (*Firmicutes*)	1.72 **a**	1.54 **a**	1.95 **a**
Gp16 (*Acidobacteria*)	1.26 **a**	1.04 **a**	1.02 **a**
Gemmatimonadetes (*Gemmatimonadetes*)	0.96 **ab**	0.82 **a**	1.01 **b**

Table S4. Identification and relative abundances of bacterial OTUs reported from SIMPER analysis and contributing ≥ 0.2% to dissimilarities in bacterial community structures among the studied layers A14 (upper), A16 (intermediate) and A18 (lower). The data represent the mean values of the six replicate libraries analyzed in each layer. For each bacterial OTU, values followed by different letters are significantly different (p≤0.05) according to Tukey's HSD test.

**OTU id**	**OTU classification at genus level (Phylum)**	**Confidence threshold (%)**	**A14**	**A16**	**A18**
OTU_1	*Pseudomonas* (*Proteobacteria*)	100	0.65 **a**	10.20 **c**	3.86 **b**
OTU_465	*Pseudomonas* (*Proteobacteria*)	100	0.95 **a**	4.07 **b**	2.86 **b**
OTU_4	*Janthinobacterium* (*Proteobacteria*)	17	0.07 **a**	0.13 **a**	2.89 **b**
OTU_2	*Jatrophihabitans* (*Actinobacteria*)	16	1.42 **a**	2.46 **a**	3.80 **b**
OTU_3	*Acidothermus* (*Actinobacteria*)	17	0.93 **a**	3.31 **b**	1.69 **a**
OTU_12	*Thermoanaerobaculum* (*Acidobacteria*)	17	1.54 **b**	0.30 **a**	0.32 **a**
OTU_3392	*Thermocatellispora* (*Actinobacteria*)	5	0.29 **a**	1.49 **b**	0.19 **a**
OTU_27	*Rhodoferax* (*Proteobacteria*)	100	0.05 **a**	0.02 **a**	1.23 **b**
OTU_9	*Pseudomonas* (*Proteobacteria*)	88	0.55 **a**	1.47 **b**	1.31 **ab**
OTU_7	*Aquihabitans* (*Actinobacteria*)	10	0.92 **a**	1.73 **b**	1.92 **b**
OTU_13	*Polaromonas* (*Proteobacteria*)	96	0.25 **a**	0.07 **a**	1.14 **b**
OTU_11	*Garicola* (*Actinobacteria*)	22	0.71 **a**	0.52 **a**	1.06 **a**
OTU_8	*Nitrospira* (*Nitrospirae*)	100	2.21 **a**	1.38 **a**	1.53 **a**
OTU_20	*Dehalococcoides* (*Chloroflexi*)	10	0.82 **a**	0.89 **a**	1.35 **a**
OTU_6	*Thermosulfurimonas* (*Thermodesulfobacteria*)	15	0.79 **a**	1.09 **b**	1.19 **b**
OTU_179	*Pseudomonas* (*Proteobacteria*)	100	0.16 **a**	0.51 **a**	0.77 **a**
OTU_19	*Aquihabitans* (*Actinobacteria*)	14	1.74 **b**	1.12 **a**	1.07 **a**
OTU_5	*Thermomarinilinea* (*Chloroflexi*)	71	1.26 **a**	1.73 **a**	1.56 **a**
OTU_21	*Nitrospira* (*Nitrospirae*)	100	0.85 **b**	0.72 **ab**	0.28 **a**
OTU_26	*Pseudomonas* (*Proteobacteria*)	100	0.03 **a**	0.77 **b**	0.20 **a**
OTU_43	*Methyloceanibacter* (*Proteobacteria*)	72	1.09 **a**	0.61 **a**	0.66 **a**
OTU_73	*Thermodesulfobium* (*Firmicutes*)	50	0.74 **b**	0.19 **a**	0.08 **a**
OTU_17	*Rubrivirga* (*Bacteroidetes*)	17	0.40 **a**	0.46 **a**	0.87 **b**
OTU_84	*Phyllobacterium* (*Proteobacteria*)	100	0.73 **a**	0.41 **a**	0.25 **a**
OTU_112	*Bradyrhizobium* (*Proteobacteria*)	64	0.66 **b**	0.35 **ab**	0.16 **a**
OTU_157	*Acetonema* (*Firmicutes*)	40	0.65 **a**	0.37 **a**	0.23 **a**
OTU_47	*Agromyces* (*Actinobacteria*)	99	0.55 **a**	0.37 **a**	0.10 **a**
OTU_24	*Nitrospira* (*Nitrospirae*)	100	0.92 **a**	0.68 **a**	0.49 **a**
OTU_33	*Hydrogenispora* (Firmicutes)	100	0.58 **b**	0.04 **a**	0.05 **a**
OTU_16	*Desulfatitalea* (*Proteobacteria*)	7	0.52 **a**	0.87 **a**	0.88 **a**
OTU_32	*Terrimicrobium* (Verrucomicrobia)	97	0.54 **b**	0.12 **a**	0.01 **a**
OTU_34	*Thermosulfidibacter* (*Aquificae*)	18	0.22 **a**	0.54 **b**	0.69 **b**
OTU_96	*Massilia* (*Proteobacteria*)	98	0.01 **a**	0.55 **b**	0.01 **a**
OTU_14	Gp7 (*Acidobacteria*)	96	0.39 **a**	0.67 **ab**	0.79 **b**
OTU_10	*Fodinicola* (*Actinobacteria*)	8	1.08 **a**	1.47 **b**	1.44 **b**
OTU_74	*Gaiella* (*Actinobacteria*)	82	0.56 **b**	0.12 **a**	0.08 **a**
OTU_221	*Alsobacter* (*Proteobacteria*)	54	0.52 **b**	0.01**a**	0.01 **a**
OTU_83	*Anaerolinea* (*Chloroflexi*)	23	0.54 **b**	0.34 **ab**	0.11 **a**
OTU_15	*Thermoanaerobaculum* (*Acidobacteria*)	6	0.57 **a**	0.46 **a**	0.62 **a**
OTU_39	*Desulfovirga* (*Proteobacteria*)	18	0.25 **a**	0.37 **a**	0.68 **b**
OTU_45	*Rhodococcus* (*Actinobacteria*)	100	0.07 **a**	0.51 **b**	0.01 **a**
OTU_28	*Hoeflea* (*Proteobacteria*)	70	0.26 **b**	0.04 **a**	0.41 **a**
OTU_38	*Nitrospira* (*Nitrospirae*)	89	0.60 **b**	0.30 **a**	0.15 **a**
OTU_51	*Phycisphaera* (*Planctomycetes*)	22	0.45 **b**	0.07 **a**	0.03 **a**
OTU_48	*Thermodesulfobium* (*Firmicutes*)	54	0.54 **a**	0.54 **a**	0.92 **b**
OTU_49	*Gaiella* (*Actinobacteria*)	95	0.51 **b**	0.14 **a**	0.17 **a**
OTU_41	*Nitrospira* (*Nitrospirae*)	98	0.04 **a**	0.35 **b**	0.46 **b**
OTU_22	Gp7 (*Acidobacteria*)	100	0.91 **a**	0.61 **a**	0.60 **a**
OTU_18	Gp15 (*Acidobacteria*)	100	0.30 **a**	0.34 **a**	0.50 **a**
OTU_40	*Gaiella* (*Actinobacteria*)	100	0.29 **a**	0.56 **b**	0.55 **b**
OTU_57	*Dongia* (*Proteobacteria*)	82	0.37 **b**	0.26 **ab**	0.08 **a**
OTU_124	*Hyphomicrobium* (*Proteobacteria*)	55	0.56 **a**	0.38 **a**	0.28 **a**
OTU_111	*Paenibacillus* (*Firmicutes*)	99	0.34 **a**	0.39 **a**	0.61 **b**
OTU_135	Gp4 (*Acidobacteria*)	100	0.40 **b**	0.18 **a**	0.09 **a**
OTU_75	*Smithella* (*Proteobacteria*)	32	0.33 **b**	0.12 **a**	0.09 **a**
OTU_4115	Gp18 (*Acidobacteria*)	96	0.32 **b**	0.04 **a**	0.02 **a**
OTU_54	*Kribbella* (*Actinobacteria*)	100	0.06 **a**	0.36 **b**	0.04 **a**
OTU_59	*Desulfobaculum* (*Proteobacteria*)	19	0.57 **b**	0.28 **a**	0.28 **a**
OTU_66	*Sulfurisoma* (*Proteobacteria*)	25	0.23 **a**	0.35 **a**	0.40 **a**
OTU_136	Gp4 (*Acidobacteria*)	100	0.38 **a**	0.29 **a**	0.39 **a**
OTU_89	Gp7 (*Acidobacteria*)	100	0.54 **b**	0.23 **a**	0.32 **a**
OTU_30	Gp7 (*Acidobacteria*)	98	0.14 **a**	0.29 **b**	0.42 **b**
OTU_177	*Massilia* (*Proteobacteria*)	100	0.04 **a**	0.10 **a**	0.34 **b**
OTU_44	*Longilinea* (*Chloroflexi*)	26	0.54 **a**	0.51 **a**	0.43 **a**
OTU_69	*Thermoflexus* (*Chloroflexi*)	22	0.17 **a**	0.29 **ab**	0.39 **b**
OTU_1468	*Gaiella* (*Actinobacteria*)	99	0.41 **b**	0.24 **ab**	0.18 **a**
OTU_97	*Thermotunica* (*Actinobacteria*)	40	0.28 **b**	0.02 **a**	0.01 **a**

Table S5. Relative abundances (%) of the archaeal phyla and the most abundant archaeal classes found in the studied layers A14 (upper), A16 (intermediate) and A18 (lower). The data represent the mean values of the six replicate libraries analyzed in each layer. For each taxonomic group, values followed by different letters are significantly different ($p \leq 0.05$) according to Tukey's HSD test.

	**A14**	**A16**	**A18**
**Archaeal phyla**			
Thaumarchaeota	90.93 **a**	88.88 **a**	89.92 **a**
Euryarchaeota	7.52 **a**	9.24 **a**	8.95 **a**
Pacearchaeota	1.27 **a**	1.58 **a**	0.89 **a**
Aenigmarchaeota	0.07 **a**	0.09 **a**	0.09 **a**
Woesearchaeota	0.01 **a**	0.10 **a**	0.04 **a**
Crenarchaeota	0.02 **a**	0.01 **a**	0.02 **a**
Unclassified	0.18 **a**	0.10 **a**	0.10 **a**
**Archaeal classes**			
Nitrososphaerales (*Thaumarchaeota*)	90.10 **a**	87.30 **a**	89.07 **a**
Thermoplasmata (*Euryarchaeota*)	6.30 **a**	7.13 **a**	6.76 **a**
Nitrosopumilales (*Thaumarchaeota*)	0.42 **a**	0.56 **a**	0.39 **a**
Pacearchaeota Incertae Sedis AR13 (*Pacearchaeota)*	1.27 **a**	1.58 **a**	0.89 **a**
Candidatus Aenigmarchaeum (*Aenigmarchaeota*)	0.07 **a**	0.09 **a**	0.09 **a**
Methanomicrobia (*Euryarchaeota*)	0.13 **a**	0.07 **a**	0.17 **a**
Thermoprotei (*Crenarchaeota*)	0.02 **a**	0.01 **a**	0.02 **a**

Table S6. Identification and relative abundances of archaeal OTUs reported from SIMPER analysis and contributing ≥ 0.2% to dissimilarities in archaeal community structures among the studied layers A14 (upper), A16 (intermediate) and A18 (lower). The data represent the mean values of the six replicate libraries analyzed in each layer. For each archaeal OTU, values followed by different letters are significantly different (p≤0.05) according to Tukey's HSD test.

OTU id	OTU classification at genus level (Phylum)	Confidence threshold (%)	A14	A16	A18
OTU_11	*Nitrososphaera* (*Thaumarchaeota*)	100	42.58 **a**	60.54 **b**	66.89 **b**
OTU_16	*Nitrososphaera* (*Thaumarchaeota*)	100	29.03 **b**	12.26 **a**	9.13 **a**
OTU_53	*Nitrososphaera* (*Thaumarchaeota*)	100	6.64 **a**	3.75 **a**	3.37 **a**
OTU_104	*Methanomassiliicoccus* (*Euryarchaeota*)	66	3.85 **a**	4.48 **a**	4.01 **a**
OTU_87	*Nitrososphaera* (*Thaumarchaeota*)	100	4.78 **a**	4.49 **a**	3.88 **a**
OTU_109	*Nitrososphaera* (*Thaumarchaeota*)	100	4.56 **a**	4.04 **a**	3.57 **a**
OTU_384	*Methanomassiliicoccus* (*Euryarchaeota*)	69	0.61 **a**	0.32 **a**	0.63 **a**
OTU_4367	*Methanomassiliicoccus* (*Euryarchaeota*)	66	0.66 **a**	0.99 **a**	0.75 **a**
OTU_368	*Methanomassiliicoccus* (*Euryarchaeota*)	57	0.58 **a**	0.77 **a**	0.73 **a**
OTU_552	*Nitrososphaera* (*Thaumarchaeota*)	49	0.38 **a**	0.95 **b**	0.38 **a**
OTU_594	*Methanomassiliicoccus* (*Euryarchaeota*)	41	0.27 **a**	0.67 **a**	0.62 **a**
OTU_410	Pacearchaeota Incertae Sedis AR13 (*Pacearchaeota*)	53	0.44 **a**	0.15 **a**	0.06 **a**
OTU_399	*Nitrososphaera* (*Thaumarchaeota*)	100	0.68 **a**	0.48 **a**	0.54 **a**
OTU_561	*Nitrososphaera* (*Thaumarchaeota*)	100	0.55 **b**	0.14 **a**	0.11 **a**
OTU_897	*Methanomassiliicoccus* (*Euryarchaeota*)	19	0.06 **a**	0.39 **a**	0.20 **a**
OTU_383	*Pacearchaeota* Incertae Sedis AR13 (*Pacearchaeota*)	64	0.38 **a**	0.13 **a**	0.08 **a**
OTU_544	*Nitrosopumilus* (*Thaumarchaeota*)	100	0.27 **a**	0.44 **a**	0.36 **a**
OTU_616	*Nitrososphaera* (*Thaumarchaeota*)	100	0.33 **a**	0.37 **a**	0.50 **a**
OTU_3467	*Methanomassiliicoccus* (*Euryarchaeota*)	37	0.11 **a**	0.25 **ab**	0.42 **b**
OTU_887	Pacearchaeota Incertae Sedis AR13 (*Pacearchaeota*)	77	0.00 **a**	0.35 **a**	0.00 **a**
OTU_529	Pacearchaeota Incertae Sedis AR13 (*Pacearchaeota*)	80	0.25 **a**	0.08 **a**	0.08 **a**
OTU_1270	*Nitrososphaera* (*Thaumarchaeota*)	100	0.39 **b**	0.20 **ab**	0.10 **a**
OTU_1426	*Pacearchaeota* Incertae Sedis AR13 (*Pacearchaeota*)	68	0.08 **a**	0.02 **a**	0.26 **a**
OTU_573	*Methanomassiliicoccus* (*Euryarchaeota*)	36	0.23 **a**	0.29 **a**	0.19 **a**
OTU_4810	*Nitrososphaera* (*Thaumarchaeota*)	100	0.32 **a**	0.30 **a**	0.45 **a**
OTU_1054	*Pacearchaeota* Incertae Sedis AR13 (*Pacearchaeota*)	67	0.01 **a**	0.25 **a**	0.01 **a**
OTU_810	*Methanomassiliicoccus* (*Euryarchaeota*)	51	0.20 **a**	0.21 **a**	0.07 **a**
OTU_1446	*Methanomassiliicoccus* (*Euryarchaeota*)	27	0.07 **a**	0.21 **a**	0.12 **a**
OTU_871	Pacearchaeota Incertae Sedis AR13 (*Pacearchaeota*)	81	0.04 **a**	0.11 **a**	0.14 **a**
OTU_1143	Pacearchaeota Incertae Sedis AR13 (*Pacearchaeota*)	97	0.10 **a**	0.06 **a**	0.12 **a**
OTU_1465	*Nitrososphaera* (*Thaumarchaeota*)	69	0.08 **a**	0.18 **a**	0.12 **a**
OTU_931	*Methanomassiliicoccus* (*Euryarchaeota*)	76	0.11 **a**	0.17 **a**	0.24 **a**
OTU_1826	Pacearchaeota Incertae Sedis AR13 (*Pacearchaeota*)	92	0.00 **a**	0.13 **a**	0.04 **a**
OTU_1372	Pacearchaeota Incertae Sedis AR13 (*Pacearchaeota*)	90	0.01 **a**	0.04 **a**	0.12 **a**
OTU_2665	*Methanomassiliicoccus* (*Euryarchaeota*)	70	0.06 **a**	0.15 **a**	0.06 **a**
OTU_1918	*Methanomassiliicoccus* (*Euryarchaeota*)	53	0.00 **a**	0.11 **a**	0.06 **a**

Table S7. Relative abundance (%) of the fungal phyla and the most abundant fungal classes found in the studied layers A14 (upper), A16 (intermediate) and A18 (lower). The data represent the mean values of the six replicate libraries analyzed in each layer. For each taxonomic group, values followed by different letters are significantly different (p≤0.05) according to Tukey's HSD test.

	**A14**	**A16**	**A18**
**Fungal phyla**			
Ascomycota	57.66 **a**	80.48 **a**	54.68 **a**
Basidiomycota	23.46 **a**	13.62 **a**	35.13 **a**
Chytridiomycota	7.71 **a**	3.43 **a**	6.09 **a**
Zygomycota	1.26 **a**	1.39 **a**	0.70 **a**
Glomeromycota	0.03 **a**	0.00 **a**	0.01 **a**
Unclassified	9.88 **a**	1.07 **a**	3.39 **a**
**Fungal classes**			
Sordariomycetes (*Ascomycota*)	28.95 **a**	71.82 **b**	42.04 **a**
Agaricomycetes (*Basidiomycota*)	19.79 **a**	13.21 **a**	32.03 **a**
Leotiomycetes (*Ascomycota*)	14.73 **b**	1.73 **a**	0.22 **a**
Dothideomycetes (*Ascomycota*)	1.33 **a**	4.47 **a**	3.32 **a**
Eurotiomycetes (*Ascomycota*)	0.74 **a**	0.53 **a**	4.46 **a**
Incertae sedis 14 (*Ascomycota*)	1.43 **a**	0.04 **a**	3.30 **a**
Incertae sedis 10 (*Zygomycota*)	1.26 **a**	1.39 **a**	0.70 **a**
Tremellomycetes (*Basidiomycota*)	1.48 **a**	0.01 **a**	0.01 **a**

Table S8. Identification and relative abundances of fungal OTUs reported from SIMPER analysis and contributing ≥ 0.2% to dissimilarities in fungal community structures among the studied layers A14 (upper), A16 (intermediate) and A18 (lower). The data represent the mean values of the six replicate libraries analyzed in each layer. For each fungal OTU, values followed by different letters are significantly different (p≤0.05) according to Tukey's HSD test.

**OTU id**	**OTU classification at genus level (Phylum)**	**Confidence threshold (%)**	**A14**	**A16**	**A18**
OTU_1	*Ilyonectria* (*Ascomycota*)	99	6.82 **a**	49.71 **b**	11.31 **a**
OTU_3	*Ilyonectria* (*Ascomycota*)	100	2.29 **a**	2.35 **a**	15.87 **b**
OTU_2	*Russulales* unidentified (*Basidiomycota*)	47	0.12 **a**	0.15 **a**	7.97 **b**
OTU_33	*Ascomycota* unidentified (*Ascomycota*)	67	7.79 **a**	0.04 **a**	0.04 **a**
OTU_5	*Tetracladium* (*Ascomycota*)	99	6.46 **b**	0.33 **a**	0.16 **a**
OTU_10	*Chytridiomycota* unidentified (*Chytridiomycota*)	25	6.01 **a**	0.74 **a**	0.08 **a**
OTU_50	Fungi unidentified (Fungi)	23	5.53 **a**	0.03 **a**	0.86 **a**
OTU_19	*Calcarisporiella* (*Ascomycota*)	97	1.39 **a**	0.06 **a**	4.34 **a**
OTU_14	*Scopuloides* (*Basidiomycota*)	99	0.06 **a**	0.04 **a**	5.26 **a**
OTU_13	*Cordyceps* (*Ascomycota*)	100	0.27 **a**	0.77 **a**	3.94 **a**
OTU_4	*Synchytrium* (*Chytridiomycota*)	25	0.78 **a**	3.60 **a**	0.68 **a**
OTU_9	*Tricholomataceae* unidentified (*Basidiomycota*)	100	2.61 **a**	0.62 **a**	1.92 **a**
OTU_21	*Davidiella* (*Ascomycota*)	96	0.70 **a**	0.34 **a**	3.07 **a**
OTU_8	*Rhexocercosporidium* (*Ascomycota*)	97	2.91 **a**	0.75 **a**	0.06 **a**
OTU_16	*Talaromyces* (*Ascomycota*)	90	0.02 **a**	0.02 **a**	2.97 **a**
OTU_17	*Ascomycota* unidentified (*Ascomycota*)	84	2.93 **b**	0.07 **a**	0.03 **a**
OTU_11	*Nectriaceae* unidentified (*Ascomycota*)	94	2.74 **a**	2.57 **a**	1.43 **a**
OTU_6	*Botryobasidium* (*Basidiomycota*)	100	2.78 **a**	0.05 **a**	0.05 **a**
OTU_20	*Vuilleminia* (*Basidiomycota*)	100	2.58 **a**	0.03 **a**	0.05 **a**
OTU_29	*Heterobasidion* (*Basidiomycota*)	100	0.06 **a**	2.34 **a**	0.03 **a**
OTU_53	*Hyphodermella* (*Basidiomycota*)	56	2.23 **a**	0.14 **a**	0.07 **a**
OTU_12	*Trametes* (*Basidiomycota*)	98	0.91 **a**	0.05 **a**	1.64 **a**
OTU_26	*Pucciniastrum* (*Basidiomycota*)	3	0.03 **a**	0.02 **a**	2.23 **a**
OTU_27	*Junghuhnia* (*Basidiomycota*)	5	2.09 **a**	0.18 **a**	0.02 **a**
OTU_23	*Baeospora* (*Basidiomycota*)	100	0.03 **a**	0.03 **a**	2.22 **a**
OTU_44	*Thelephoraceae* unidentified (*Basidiomycota*)	10	2.19 **a**	0.09 **a**	0.04 **a**
OTU_57	*Mortierella* (Zygomycota)	16	2.16 **a**	0.03 **a**	0.01 **a**
OTU_32	*Hypocreales* unidentified (*Ascomycota*)	96	0.66 **a**	0.02 **a**	1.59 **a**
OTU_24	*Agaricales* unidentified (*Basidiomycota*)	87	0.02 **a**	2.09 **a**	0.04 **a**
OTU_38	*Tremellales* unidentified (*Basidiomycota*)	1	1.96 **a**	0.01 **a**	0.02 **a**
OTU_18	Fungi unidentified (Fungi)	50	0.04 **a**	0.03 **a**	1.93 **a**
OTU_28	*Ceratobasidium* (*Basidiomycota*)	99	0.02 **a**	0.02 **a**	1.89 **a**
OTU_43	*Davidiella* (*Ascomycota*)	100	0.04 **a**	1.55 **b**	0.65 **ab**
OTU_41	*Mortierella* (Zygomycota)	78	0.46 **a**	1.61 **a**	0.10 **a**
OTU_47	*Beauveria* (*Ascomycota*)	57	0.02 **a**	0.06 **a**	1.75 **a**
OTU_36	*Rhodotorula* (*Basidiomycota*)	100	0.66 **a**	0.36 **a**	1.09 **a**
OTU_40	*Antrodia* (*Basidiomycota*)	100	0.02 **a**	0.02 **a**	1.77 **a**
OTU_31	*Ophiocordyceps* (*Ascomycota*)	22	1.76 **a**	0.01 **a**	0.02 **a**
OTU_60	*Skeletocutis* (*Basidiomycota*)	100	0.04 **a**	0.01 **a**	1.69 **a**
OTU_15	*Phellinus* (*Basidiomycota*)	96	1.68 **a**	0.02 **a**	0.03 **a**
OTU_34	*Alternaria* (*Ascomycota*)	97	0.04 **a**	1.04 **a**	0.84 **a**
OTU_54	*Myxotrichaceae* unidentified (*Ascomycota*)	93	0.94 **a**	0.02 **a**	0.86 **a**
OTU_59	*Heterobasidion* (*Basidiomycota*)	100	1.27 **a**	0.38 **a**	0.03 **a**
OTU_46	*Aspergillus* (*Ascomycota*)	100	1.57 **a**	0.02 **a**	0.03 **a**
OTU_7	*Fomitopsis* (*Basidiomycota*)	100	0.06 **a**	0.28 **a**	1.41 **a**
OTU_37	*Ramicandelaber* (Zygomycota)	34	1.45 **b**	0.25 **a**	0.03 **a**
OTU_42	*Agaricaceae* unidentified (*Basidiomycota*)	9	0.03 **a**	0.01 **a**	1.50 **a**
OTU_85	*Cryptococcus* (*Basidiomycota*)	100	1.49 **a**	0.02**a**	0.02 **a**
OTU_70	Fungi unidentified (Fungi unidentified)	20	0.02 **a**	0.01**a**	1.47 **a**
OTU_30	*Auriscalpium* (*Basidiomycota*)	100	0.02 **a**	1.41 **a**	0.03 **a**
OTU_45	*Ischnoderma* (*Basidiomycota*)	100	1.42 **a**	0.03 **a**	0.05 **a**
OTU_64	*Resinicium* (*Basidiomycota*)	100	1.31 **a**	0.17 **a**	0.03 **a**
OTU_39	*Agaricomycetes* unidentified (*Basidiomycota*)	15	0.02 **a**	1.40 **a**	0.03**a**
OTU_61	*Rasamsonia* (*Ascomycota*)	100	0.03 **a**	0.01 **a**	1.39 **a**
OTU_22	*Alternaria* (*Ascomycota*)	100	0.03 **a**	1.39 **a**	0.04 **a**

OTU_52	*Ascomycota* unidentified (*Ascomycota*)	95	0.21 **a**	1.23 **a**	0.03 **a**
OTU_48	*Ilyonectria* (*Ascomycota*)	98	0.12 **a**	0.55 **a**	1.20 **a**
OTU_51	*Mortierella* (Zygomycota)	99	0.38 **a**	0.12 **a**	1.09 **a**
OTU_56	*Inocybe* (*Basidiomycota*)	10	0.60 **a**	0.88 **a**	0.02 **a**
OTU_58	*Devriesia* (*Ascomycota*)	98	1.33 **a**	0.01 **a**	0.01 **a**
OTU_49	*Ascomycota* unidentified (*Ascomycota*)	48	1.34 **b**	0.02 **a**	0.02 **a**
OTU_62	*Agaricomycetes* unidentified (*Basidiomycota*)	100	0.03 **a**	1.23 **a**	0.04 **a**
OTU_55	Fungi unidentified (Fungi)	11	0.03 **a**	1.22 **a**	0.02 **a**
OTU_76	*Ophiocordyceps* (*Ascomycota*)	14	0.01 **a**	0.01 **a**	1.17 **a**
OTU_35	*Acremonium* (*Ascomycota*)	90	1.31 **a**	0.27 **a**	0.29 **a**
OTU_67	*Phellodon* (*Basidiomycota*)	5	0.01 **a**	1.09 **a**	0.01 **a**
OTU_63	*Polyporaceae* unidentified (*Basidiomycota*)	100	0.03 **a**	1.06 **a**	0.04 **a**
OTU_66	*Candida* (*Ascomycota*)	100	0.02 **a**	1.07 **a**	0.03 **a**
OTU_68	*Agaricales* unidentified (*Basidiomycota*)	100	0.01 **a**	1.05 **a**	0.01 **a**
OTU_65	*Phaeococcomyces* (*Ascomycota*)	100	0.01 **a**	0.01 **a**	1.04 **a**
OTU_69	*Septoglomus* (Glomeromycota)	7	0.26 **a**	0.08 **a**	0.87 **a**
OTU_74	*Rhodotorula* (*Basidiomycota*)	1	0.98 **a**	0.01 **a**	0.00 **a**
OTU_87	*Armillaria* (*Basidiomycota*)	100	0.49 **a**	0.55 **a**	0.02 **a**
OTU_77	*Sistotrema* (*Basidiomycota*)	51	0.02 **a**	0.95 **a**	0.02 **a**
OTU_73	*Tremellales* unidentified (*Basidiomycota*)	16	0.90 **a**	0.02 **a**	0.02 **a**
OTU_71	*Ramicandelaber* (Zygomycota)	97	0.55 **a**	0.44 **a**	0.01**a**
OTU_78	*Conocybe* (*Basidiomycota*)	100	0.01 **a**	0.02 **a**	0.79 **a**
OTU_72	*Mortierella* (Zygomycota)	100	0.79 **a**	0.09 **a**	0.19 **a**
OTU_75	*Tubulicrinis* (*Basidiomycota*)	100	0.01 **a**	0.79 **a**	0.02 **a**
OTU_79	Fungi unidentified (Fungi)	17	0.74 **a**	0.02 **a**	0.02 **a**
OTU_25	*Fusarium* (*Ascomycota*)	93	0.91 **a**	1.26 **a**	0.83 **a**
OTU_89	*Tomentella* (*Basidiomycota*)	99	0.02 **a**	0.01 **a**	0.70 **a**
OTU_80	*Ascomycota* unidentified (*Ascomycota*)	100	0.01 **a**	0.01 **a**	0.70 **a**
OTU_84	*Boletus* (*Basidiomycota*)	83	0.01 **a**	0.64 **a**	0.01 **a**
OTU_98	*Mortierella* (Zygomycota)	100	0.60 **a**	0.06 **a**	0.14 **a**
OTU_81	*Phoma* (*Ascomycota*)	99	0.03 **a**	0.08 **a**	0.57 **a**
OTU_82	*Taphrina* (*Ascomycota*)	89	0.02 **a**	0.61 **a**	0.02 **a**
OTU_88	*Knufia* (*Ascomycota*)	7	0.01 **a**	0.09 **a**	0.53 **a**
OTU_83	*Basidiomycota* unidentified (*Basidiomycota*)	47	0.57 **a**	0.00 **a**	0.03 **a**
OTU_86	*Datronia* (*Basidiomycota*)	100	0.01 **a**	0.00 **a**	0.55 **a**
OTU_90	*Aspergillus* (*Ascomycota*)	100	0.01 **a**	0.52 **a**	0.00 **a**
OTU_94	*Penicillium* (*Ascomycota*)	100	0.01 **a**	0.24 **a**	0.31 **a**

Table S9. Results of pairwise PERMANOVA (permutational analysis of variance) and ANOSIM (analysis of similarities) comparing OTU-based bacterial and fungal community structures found in the layers A14 (upper), A16 (intermediate) and A18 (lower) (present study) and at the locations A2, A3, A6 and A7 (studied in a previous work[5] and also situated at Monte Iato settlement). F and R values in bold denote statistical significance ($p \leq 0.05$).

	**PERMANOVA**				**ANOSIM**			
Bacterial communities								
	**A2**	**A3**	**A6**	**A7**	**A2**	**A3**	**A6**	**A7**
**A14**	**5.380** (0.0355)	**4.859** (0.0328)	**4.645** (0.0342)	**3.929** (0.0374)	**0.750** (0.0354)	**0.750** (0.0319)	**0.844** (0.0332)	0.260 (0.1519)
**A16**	**15.280** (0.0381)	**14.930** (0.0358)	**15.260** (0.0375)	**13.780** (0.0367)	**1.000** (0.0353)	**1.000** (0.0355)	**1** (0.0352)	**1** (0.0376)
**A18**	**19.580** (0.0357)	**20.000** (0.0412)	**20.840** (0.0342)	**19.480** (0.0353)	**1.000** (0.0377)	**1** (0.0322)	**1** (0.0396)	**1** (0.0381)
Fungal communities								
	**A2**	**A3**	**A6**	**A7**	**A2**	**A3**	**A6**	**A7**
**A14**	**3.416** (0.0031)	**2.923** (0.0376)	**3.279** (0.0353)	**3.471** (0.0355)	**0.655** (0.0024)	0.490 (0.1054)	**1** (0.0379)	**0.771** (0.0360)
**A16**	**5.896** (0.0028)	**6.128** (0.0375)	**6.866** (0.0396)	**7.623** (0.0377)	**0.857** (0.0020)	**1** (0.0376)	**1** (0.0329)	**1** (0.0359)
**A18**	**2.842** (0.0022)	**2.231** (0.0345)	**2.649** (0.0403)	**2.914** (0.0345)	**0.585** (0.0025)	0.250 (0.2523)	**1** (0.0380)	**1** (0.0343)

Table S10. Relative abundances (%) of the bacterial phyla found in the layers A14 (upper), A16 (intermediate) and A18 (lower) (present study) and at the locations A2, A3, A6 and A7 (studied in a previous work[5] and also situated at Monte Iato settlement). For each taxonomic group, values followed by different letters are significantly different (p≤0.05) according to Tukey's HSD test.

	**A14**	**A16**	**A18**	**A2**	**A3**	**A6**	**A7**
Proteobacteria	21.44 **b**	30.08 **c**	27.30 **c**	10.97 **a**	13.71 **a**	12.03 **a**	16.36 **ab**
Actinobacteria	20.26 **a**	24.14 **abc**	21.66 **ab**	30.10 **cd**	28.02 **bc**	42.11 **e**	37.60 **de**
Acidobacteria	15.37 **b**	11.15 **a**	12.45 **a**	15.26 **b**	16.83 **b**	10.14 **a**	10.15 **a**
Firmicutes	6.65 **b**	4.47 **ab**	5.45 **b**	0.38 **a**	0.39**a**	0.27 **a**	0.32 **a**
Chloroflexi	4.69 **b**	4.74 **b**	4.64 **b**	3.07 **a**	2.24 **a**	2.98 **a**	2.62 **a**
Nitrospirae	5.51 **d**	4.31 **c**	3.99 **bc**	2.78 **ab**	2.42 **a**	2.31 **a**	3.91 **abc**
Planctomycetes	3.83 **c**	2.56 **a**	2.64 **ab**	5.50 **d**	7.57 **e**	4.19 **bcd**	2.87 **abc**
Others	5.44 **abc**	3.00 **a**	3.55 **a**	5.16 **abc**	3.97 **ab**	8.92 **bc**	9.34 **c**
Unclassified	16.80 **a**	15.56 **a**	18.31 **ab**	26.79 **c**	24.85 **bc**	17.04 **ab**	16.83 **ab**

Table S11. Relative abundances (%) of the fungal classes found in the layers A14 (upper), A16 (intermediate) and A18 (lower) (present study) and at the locations A2, A3, A6 and A7 (studied in a previous work[5] and also situated at Monte Iato settlement). For each taxonomic group, values followed by different letters are significantly different (p≤0.05) according to Tukey's HSD test.

	**A14**	**A16**	**A18**	**A2**	**A3**	**A6**	**A7**
Sordariomycetes (*Ascomycota*)	28.95 **c**	71.82 **d**	42.04 **c**	19.69 **b**	25.30 **b**	3.86 **a**	9.21 **a**
Agaricomycetes (*Basidiomycota*)	19.79 **b**	13.21 **b**	32.03 **b**	0.83 **a**	1.19 **a**	3.62 **a**	3.18 **a**
Leotiomycetes (*Ascomycota*)	14.73 **b**	1.73 **a**	0.22 **a**	0.51 **a**	8.82 **ab**	0.04 **a**	1.63 **a**
Dothideomycetes (*Ascomycota*)	1.33 **a**	4.47 **a**	3.32 **a**	53.91 **b**	47.36 **b**	0.40 **a**	0.35 **a**
Eurotiomycetes (*Ascomycota*)	0.74 **a**	0.53 **a**	4.46 **a**	0.50 **a**	1.45 **a**	0.62 **a**	4.01 **a**
Incertae_sedis_10 (Zygomycota)	1.26 **a**	1.39 **a**	0.70 **a**	9.31 **a**	4.06 **a**	0.13 **a**	0.81 **a**
Pezizomycetes (*Ascomycota*)	0.00 **a**	0.02 **a**	0.00 **a**	0.69 **a**	0.37 **a**	24.05 **b**	45.52 **c**
Others	3.49 **a**	1.10 **a**	4.41 **a**	0.19 **a**	1.75 **a**	1.41 **a**	0.17 **a**
Unclassified	29.70 **ab**	5.73 **a**	12.82 **a**	14.36 **ab**	9.71 **ab**	65.88 **b**	35.12 **ab**
